# A Bayesian Spatiotemporal Model for Joint Estimation of Brain Activation and Connectivity in fMRI Studies

**DOI:** 10.1002/sim.70712

**Published:** 2026-08-25

**Authors:** Parisa Naseri, Yuliya Shapovalova, Ioan Gabriel Bucur, Charlotte Cambier van Nooten, Giancarlo Valente, Tom Heskes

**Affiliations:** ^1^ Institute for Computing and Information Sciences Radboud University Nijmegen the Netherlands; ^2^ Faculty of Psychology and Neuroscience Maastricht University Maastricht the Netherlands

**Keywords:** Bayesian inference, brain activation, brain connectivity, functional magnetic resonance imaging, spatiotemporal modeling

## Abstract

Task‐based functional magnetic resonance imaging (fMRI) experiments play a crucial role in modern data‐driven neuroscience research. These studies often aim to understand how external stimuli trigger activation in specific brain regions and to explore functional connectivity patterns among predefined regions, commonly referred to as regions of interest (ROIs). Accurately estimating both brain activation and inter‐regional connectivity is challenging due to complex spatiotemporal correlations and low signal‐to‐noise ratios inherent in fMRI data. This paper introduces a joint spatiotemporal Bayesian framework that simultaneously models activation and connectivity across multiple subjects while estimating the hemodynamic response function (HRF) for each region. Spatial dependencies are captured via an unweighted graph‐Laplacian prior on regression and autoregressive coefficients, and region‐specific random effects are modeled using a Bayesian Gaussian graphical model to reflect connectivity among ROIs. We evaluate the performance of the model through simulation studies, demonstrating robust estimation under realistic low signal‐to‐noise conditions. The approach is then applied to a multisubject motor task dataset from the Human Connectome Project (HCP), mapping brain motor areas associated with specific movements (e.g., finger, toe, tongue) and assessing their activation and lateralization in response to visual cues. The model is further evaluated on the Individual Brain Charting (IBC) dataset, a high‐resolution 3T fMRI dataset designed for fine‐grained cognitive mapping across multiple tasks. Finally, the model is validated through comparisons with the classical general linear model (GLM), which is commonly used in the neuroscience community, highlighting the advantages of our Bayesian approach in jointly capturing activation and connectivity patterns.

## 1 Introduction

Functional magnetic resonance imaging (fMRI) is a key tool in cognitive neuroscience, providing a noninvasive method to study brain activity through the measurement of blood oxygen level‐dependent (BOLD) signals. This technique enables researchers to explore how different brain regions engage during cognitive or sensorimotor tasks and how they communicate with one another. These are commonly referred to as brain activation and connectivity, respectively. Activation identifies which regions are involved in a specific task, while connectivity refers to functional interactions between different ROIs, anatomical connections, or effective connectivity [1, 2]. The BOLD signal is obtained from every location in the brain, captured in voxels, which partition the brain into equally sized boxes [3]. The shape of the BOLD signal depends on the stimulus and hemodynamic response function (HRF), which describes the hemodynamic response to neuronal events and changes across the brain regions and subjects [4]. As neuronal activity is indirectly measured through the hemodynamic response, it is important to correctly estimate the HRF for accurate inference of neural activation [4, 5].

In conventional methods, activation is typically estimated using a general linear model (GLM), where task onsets convolved with a canonical HRF form the predictors of the BOLD signal in a linear regression framework. The resulting coefficients quantify task‐related activation in different regions. Functional connectivity is then assessed in a separate step, often based on the residuals of the activation model. This two‐stage approach, however, fails to account for the uncertainty introduced by activation estimates and ignores important spatial and temporal correlations inherent in fMRI data [6]. Therefore, a unified modeling approach that accounts for the complex spatial and temporal structures and jointly estimates connectivity will improve the power to correctly identify important activation and provide more precise inference on connectivity patterns [7].

### 1.1 Overview of Related Work

Various Bayesian models have been developed that incorporate spatial and temporal structure into the analysis of brain activation [8, 9, 10, 11, 12, 13, 14, 15, 16]. These models improve activation detection by using priors that reflect anatomical or functional adjacency and by modeling temporal dependencies through autoregressive processes. However, many such models treat activation and connectivity as separate inferential problems, and often fix the HRF between subjects and regions, despite recent evidence of substantial spatial heterogeneity in the hemodynamic response across the brain [17].

A closely related line of work, known as Joint Detection‐Estimation (JDE), has addressed the simultaneous estimation of brain activation and region‐specific hemodynamic response functions within a fully Bayesian framework [18]. Early work by Makni et al. [19] introduced a hierarchical model that jointly estimates activation effects and parcel‐level HRFs using spatial priors on activation states and nonparametric HRF modeling. This line of research directly tackles the coupling between neuronal activation and hemodynamic variability that motivates our approach. Subsequent developments introduced spatially adaptive mixture models for activation detection within the JDE framework using Markov random field priors, which enforce spatial coherence in activation maps [20]. In addition, between‐subject and between‐region variability in the hemodynamic response has also been studied within the JDE framework, where Bayesian hierarchical models were used to quantify how HRF variability impacts both activation estimation and group‐level inference [21].

In the context of connectivity estimation, Bayesian approaches have also become increasingly popular, offering flexible ways to capture both functional and effective connectivity [8, 22, 23, 24, 25]. However, most connectivity frameworks are developed separately from activation analyses or rely on multistage procedures that may obscure the true underlying neural mechanisms. For instance, Luo [26] estimated the connectivity via a two‐stage approach that uses the residual from the activity analysis to estimate connectivity.

A more unified modeling framework integrating both activation and connectivity estimation while incorporating region‐specific HRFs and spatiotemporal dependencies can yield more accurate and interpretable insights regarding brain function. David et al. [22] and Bowman et al. [27] estimated both activation and connectivity in their methods, but relied on the conventional two‐stage approach. Other models improved on the integration of activation and connectivity, but involved simplifying assumptions, such as fixed HRFs or limited modeling of spatial and temporal dependencies. For example, Kang et al. [28] modeled activation and spatial correlations using fixed and random effects and assumed a fixed HRF. Spencer et al. [29] recently proposed a joint Bayesian additive mixed‐effect model that simultaneously incorporates brain activation at the voxel level and connectivity patterns from multiple subjects. They imposed a Gaussian graphical prior for estimation of connectivity. However, they did not estimate the HRF, and they did not take into account the spatiotemporally dependent error structures. Finally, Miranda and Morris [7] proposed a new Bayesian approach for analyzing task fMRI data that simultaneously detects activation and connectivity. Their modeling involves a hybrid tensor spatial‐temporal basis strategy that enables scalable computing, although hemodynamic response variability across subjects and regions is not explicitly addressed.

### 1.2 Proposed Approach

In the current study, we propose a novel Bayesian spatiotemporal model for the joint estimation of brain activation and connectivity in task‐based fMRI data. Our approach integrates key features from existing methods into a single framework. We build upon the general linear model‐autoregressive (GLM‐AR) framework commonly used in the Statistical Parametric Mapping (SPM) software, [30, 31, 32], extending it in several ways. We incorporate region‐specific parametric HRFs modeled as mixtures of gamma functions [33, 34, 35]. Temporal correlations are captured through region‐specific autoregressive error terms, with spatial smoothing enforced via Gaussian Markov random field (GMRF) priors on the autoregressive coefficients. To model functional connectivity, we introduce subject‐ and ROI‐specific random effects, imposing a Gaussian graphical prior with shrinkage on the precision matrix to estimate connections between regions [36]. This unified framework allows for simultaneous inference of activation and connectivity while respecting the complex spatial and temporal structures in fMRI data. We evaluate our model using simulated data under different signal‐to‐noise (SNR) levels and implement it on task‐based fMRI data from the Human Connectome Project (HCP) and the Individual Brain Charting (IBC), comparing its performance against the standard classical GLM.

The remainder of the paper is organized as follows. In Section 2, we present the model, prior distributions, and inferential procedures. Section 3 describes simulation studies to assess the performance of the model. Section 4 presents the results from the simulation studies. Section 5 introduces the two real‐world fMRI datasets and presents the results from our Bayesian model. We conclude and propose directions for future research in Section 6.

## 2 Methodology

In this section, we outline the core components of the proposed Bayesian model and describe the approach used for parameter inference. The model is designed to capture fMRI signals across multiple subjects and brain regions, and consists of several key elements. A spatial prior is placed over ROIs on both the regression coefficients and the autoregressive parameters. In addition, the hemodynamic response is modeled using a (canonical) double gamma HRF. To capture functional connectivity between brain regions, a Bayesian Lasso prior is employed. Parameter inference is performed jointly over the entire model using Markov chain Monte Carlo (MCMC) techniques. In the following sections, we explain each of these components in more detail.

### 2.1 Model Framework

We aim to model the fMRI signal across multiple subjects s=1,…,S and ROIs r=1,…,R, observed at discrete time points t=1,…,T. Activation and connectivity are modelled at the level of predefined ROIs; the BOLD signal is averaged across voxels within each ROI to yield a single regional time series per subject. Throughout this paper, matrices are denoted by bold uppercase letters, vectors by bold lowercase letters, and scalars by standard lowercase letters. However, note that while we generally denote matrices by uppercase letters, in Equation (1), the regression coefficient matrix $\beta^{s}$ is represented with a lowercase Greek letter for consistency with prior literature. The observed fMRI signal for subject s, region r, denoted by $y_{\cdot ,r}^{s}$ can typically be described using a GLM as follows:

(1) $$y_{\cdot ,r}^{s}=X_{r,\cdot }^{s}\;\beta_{\cdot ,r}^{s}+d_{r}^{s}1+e_{\cdot ,r}^{s},$$

where $y_{\cdot ,r}^{s}$ is the T×1 vector of observed BOLD responses at region r for subject s, and $X_{r,\cdot }^{s}$ is a matrix of dimension T×K which contains the values of K regressors at T time points for region r and subject s, and represents the expected shape of BOLD response. Note that the design matrix $X_{r,\cdot }^{s}$ is region‐specific, as we do not assume the same HRF across regions. The coefficients $\beta_{\cdot ,r}^{s}$ form a K×1 vector of regression parameters for region r and subject s, representing the amplitude of the BOLD response and indirectly reflecting the strength of neuronal activity. In the model, functional connectivity is represented by $d_{r}^{s}\in \mathbb{R}$, which denotes the region‐ and subject‐specific random effect. This term is the same for all time points, so it is multiplied by a T×1 column unit vector. These effects are jointly modeled to borrow information across ROIs. Finally, $e_{\cdot ,r}^{s}$ is a T×1 error vector.

**FIGURE 1:**
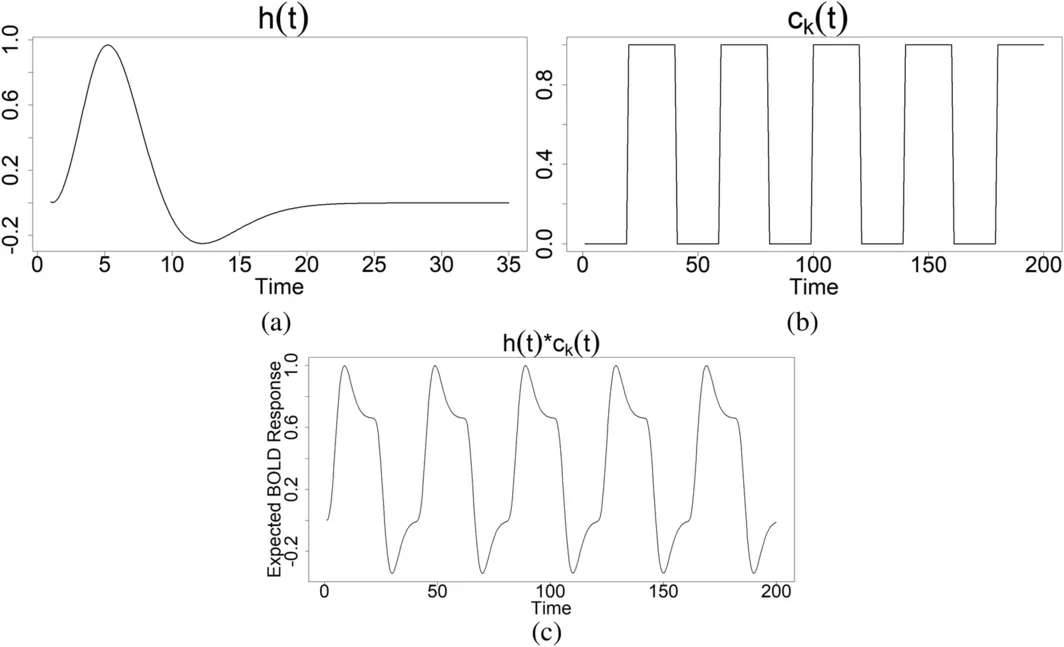
General description of the BOLD response, where the time on the x‐axis is measured in seconds. (a) Canonical double gamma HRF, (b) Stimulus indicator function, (c) Convolution of the HRF and stimulus indicator.

The expected shape of the BOLD response $X_{r,\cdot }^{s}$ is modeled as the convolution between the double gamma HRF $h_{r}^{s}(t)$ and a binary condition indicator $c_{k}(t)$, which is equal to 1 when the condition is on and to 0 otherwise. For simplicity, we consider the same HRF across subjects. Figure 1 illustrates an example HRF (Figure 1a), stimulus (Figure 1b) and corresponding convolution (Figure 1c).

For notational simplicity when writing the likelihood, we redefine Equation (1) by adding a unit vector column to $X_{r,\cdot }^{s}$ and combining $\beta_{\cdot ,r}^{s}$ and $d_{r}^{s}$ into a single matrix. We use an asterisk symbol to denote the augmented design matrix $X_{r,\cdot }^{s\;(*)}≜(X_{r,\cdot }^{s},1)$ and the augmented parameter vector $\beta_{\cdot ,r}^{s\;(*)}≜(\beta_{\cdot ,r}^{s},d_{r}^{s})$, which then become a T×(K+1) matrix and a (K+1)×1 vector, respectively. Furthermore, the errors are modeled using an autoregressive (AR) process. The overall model can be written as

(2) $$y_{\cdot ,r}^{s}=X_{r,\cdot }^{s\;(*)}\;\beta_{\cdot ,r}^{s\;(*)}+e_{\cdot ,r}^{s},$$

(3) $$e_{\cdot ,r}^{s}={\tilde{E}}_{r,\cdot }^{s}\;a_{\cdot ,r}^{s}+z_{\cdot ,r}^{s}.$$

where at the $r^{th}$ region and $s^{th}$ subject, $a_{\cdot ,r}^{s}$ is a P×1 vector of autoregressive coefficients, where P denotes the maximum order of the AR process at every region. In Equation (3), $z_{\cdot ,r}^{s}∼𝒩(0,{\left(\lambda_{r}^{s}\right)}^{-1})$ is a vector of zero‐mean Gaussian random variables each having precision $\lambda_{r}^{s}$, while ${\tilde{E}}_{r,\cdot }^{s}$ is a T×P matrix of lagged prediction errors for subject s and region r and can be rewritten as ${\tilde{e}}_{tr,\cdot }^{s}={\tilde{y}}_{tr,\cdot }^{s}-{\tilde{X}}_{tr,\cdot }^{s\;(*)}\beta_{\cdot ,r}^{s\;(*)}$, where ${\tilde{X}}_{tr,\cdot }^{s\;(*)}$ is a P×(K+1) matrix containing the P rows of the design matrix prior to time point t for subject s and region r. Similarly, ${\tilde{y}}_{tr,\cdot }^{s}$ is a P×1 vector and contains the p values of the observed BOLD responses just before time point t. More details regarding the notation can be found in Section 2 of Penny et al. [30] Equations (2) and (3) give the likelihood:

(4) $$\begin{array}{cc} & p\left(Y|\beta ,A,\lambda \right)\propto \overset{S}{\underset{s=1}{\prod }}\overset{T}{\underset{t=P+1}{\prod }}\overset{R}{\underset{r=1}{\prod }}𝒩\\ & \;\left(y_{tr}^{s}-{\left(x_{tr,\cdot }^{s\;(*)}\right)}^{⊤}\beta_{\cdot ,r}^{s\;(*)};{\left({\tilde{y}}_{tr,\cdot }^{s}-{\tilde{X}}_{tr,\cdot }^{s\;(*)}\;\beta_{\cdot ,r}^{s\;(*)}\right)}^{⊤}a_{\cdot ,r}^{s},\frac{1}{\lambda_{r}^{s}}\right), \end{array}$$

where $x_{tr,\cdot }^{s\;(*)}$ is a (K+1)×1 vector representing the $t^{th}$ row of the design matrix $X_{r,\cdot }^{s\;(*)}$, while A collects all the autoregressive coefficients and λ collects all the noise precision terms. We use the notation $M^{⊤}$ to denote the transpose of the matrix M. Note that for simplicity, our model is specified conditionally on the first P observations at each region and subject so that the likelihood function is constructed on the basis of the model for the remaining T−P observations in the time series.

### 2.2 Spatial Prior

At the next level, for each subject s, we specify a spatial smoothing prior for the regression coefficient matrix $\beta^{s}$, which is a K×R matrix with $\beta_{k,\cdot }^{s}$ denoting the $k^{th}$ row. Following Penny et al. [31] we assume that the prior for $\beta_{k,\cdot }^{s}$ takes the form

(5) $$\beta_{k,\cdot }^{s}∼𝒩(0,{\left(\alpha_{k}^{s}Q_{\beta }^{s}\right)}^{-1}),$$

where $Q_{\beta }^{s}$ is a fixed spatial R×R precision matrix and $\alpha^{s}={\left(\alpha_{1}^{s},\alpha_{2}^{s},\ldots ,\alpha_{K}^{s}\right)}^{⊤}$ are hyperparameters to be estimated from the data. We considered both to be the same for all subjects, and therefore we ignore the index s for simplicity. There are several possible choices for $Q_{\beta }^{s}$, but we will here focus on the SPM12 default choice [37], namely the Unweighted Graph‐Laplacian (UGL), which for each region has the number of adjacent regions on the diagonal and $Q_{\beta }^{s}(i,j)=-1$ if i and j are adjacent. We consider the same prior over the autoregressive coefficient vector $a_{p,\cdot }^{s}$ (the $p^{th}$ row of A) as follows

(6) $$a_{p,\cdot }^{s}∼𝒩(0,{\left(\tau_{p}^{s}Q_{a}^{s}\right)}^{-1}),$$

where $\tau^{s}={\left(\tau_{1}^{s},\tau_{2}^{s},\ldots ,\tau_{P}^{s}\right)}^{⊤}$ are hyperparameters that control how much the field can vary between neighboring regions, where large values of $\tau_{p}^{s}$ enforce a field that is spatially smooth. The precision matrix $Q_{a}^{s}$, also of size R×R, is constructed in the same way as $Q_{\beta }^{s}$.

The hyperparameters $\alpha_{k}^{s}$ and $\tau_{p}^{s}$, as well as the noise precision parameter $\lambda_{r}^{s}$, are assigned conditionally conjugate gamma hyperprior distributions: $\alpha_{k}^{s}∼𝒢(q_{2},q_{1})$, $\tau_{p}^{s}∼𝒢(v_{2},v_{1})$ and $\lambda_{r}^{s}∼𝒢(u_{2},u_{1})$, where $q_{2},v_{2},u_{2}$ are shape parameters and $q_{1},v_{1},u_{1}$ are scale parameters of the gamma distributions. The values of the prior parameters are set to $q_{1}=u_{1}=10$, $v_{1}=10,000$ and $q_{2}=v_{2}=u_{2}=0.1$ which yield a weakly informative prior, corresponding to $𝔼[\alpha_{k}^{s}]=q_{1}q_{2}=1$ with $\mathrm{Var}(\alpha_{k}^{s})=q_{1}^{2}q_{2}=10$, $𝔼[\tau_{p}^{s}]=v_{1}v_{2}=1000$ with $\mathrm{Var}(\tau_{p}^{s})=v_{1}^{2}v_{2}=10^{7}$, and $𝔼[\lambda_{r}^{s}]=u_{1}u_{2}=1$ with $\mathrm{Var}(\lambda_{r}^{s})=u_{1}^{2}u_{2}=10$.

### 2.3 Prior for HRF

In this section, we consider the canonical double gamma HRF, which is the popular choice in the neuroscience community [4]. The form of the function is, $h_{r}(t)={\left(\frac{t}{m_{1r}b_{1}}\right)}^{m_{1r}}e^{-\frac{t-m_{1r}b_{1}}{b_{1}}}-c{\left(\frac{t}{m_{2}b_{2}}\right)}^{m_{2}}e^{-\frac{t-m_{2}b_{2}}{b_{2}}}$, where $m_{1r}$ and $m_{2}$ model the delay of the response and the undershoot relative to the onset, $b_{1}$ and $b_{2}$ model the dispersion of the response and the undershoot, c models the scale of the undershoot [33]. Note that the delay of the response $m_{1r}$ can vary by region; we collect the delay parameters for all regions in $m_{1}$. We set a uniform prior distribution on the delay parameter, $m_{1r}∼U(n_{1},n_{2})$, which is allowed to be different across regions. Vague specifications can be adopted for HRF parameters, so we select a uniform prior with $n_{1}=3,n_{2}=6$, which is a reasonable range for the $m_{1}$ parameter of the double gamma HRF [33, 34, 38]. We fix the rest of the HRF parameters at the default values suggested by Glover [34]: $m_{2}=12$, $b_{1}=b_{2}=0.9$, c=0.35.

### 2.4 Bayesian Graphical Lasso Prior for Modeling Connectivity

Following Spencer et al. [29] we include connectivity between brain regions via the random effect $d_{r}^{s}$, over which we impose the Gaussian graphical Lasso prior [36]. Hence, for the vector $d^{s}$, the prior is defined as:

(7) $$\begin{array}{l} d^{s}={\left(d_{1}^{s},d_{2}^{s},\ldots ,d_{R}^{s}\right)}^{⊤}∼𝒩\left(0,\Omega^{-1}\right),\;s=1,2,\ldots ,S \end{array}$$

(8) $$\begin{array}{l} p(\Omega |\zeta )=C^{-1}\underset{k<l}{\prod }\left[\text{DE}\left(\omega_{kl};\zeta \right)\right]\overset{R}{\underset{k=1}{\prod }}\left[\mathrm{Exp}\left(\omega_{kk};\frac{\zeta }{2}\right)\right]1_{\Omega \in {𝒫}^{+}}, \end{array}$$

where, ${𝒫}^{+}$ denotes the set of symmetric positive definite matrices, and C is a normalization constant. The matrix Ω represents the precision matrix, parameterized by the vector $\Omega =(\omega_{kl}:k\leq l)$ containing its upper triangular and diagonal elements. A smaller value of $\omega_{kl}$ suggests weaker connectivity between regions k and l, while $\omega_{kl}=0$ for k<l implies conditional independence between these regions, given all others. The off‐diagonal elements of Ω follow a double exponential distribution (DE) with inverse scale (rate) ζ, whereas the diagonal elements are modeled using an exponential distribution (Exp) with rate $\frac{\zeta }{2}$.

The double exponential distribution can be written as a scale mixture of normals. Considering $\eta =(\eta_{kl}:k<l)$ as a set of latent scale parameters, we can write

(9) $$\begin{array}{l} p(\Omega |\zeta )=\int p(\Omega |\zeta ,\eta )p(\eta |\zeta )d\eta , \end{array}$$

with p(Ω|ζ,η) given by

(10) $$\begin{array}{l} p(\Omega |\zeta ,\eta )=C_{\eta }^{-1}\underset{k<l}{\prod }\left[𝒩\left(\omega_{kl};0,\eta_{kl}\right)\right]\overset{R}{\underset{k=1}{\prod }}\left[\text{Exp}\left(\omega_{kk};\frac{\zeta }{2}\right)\right]1_{\Omega \in {𝒫}^{+}}, \end{array}$$

where $C_{\eta }$ is the normalization constant, which is analytically intractable. The mixing density of η

(11) $$\begin{array}{l} p(\eta |\zeta )\propto C_{\eta }\underset{k<l}{\prod }\left[\text{Exp}\left(\eta_{kl};\frac{\zeta^{2}}{2}\right)\right]. \end{array}$$

**FIGURE 2:**
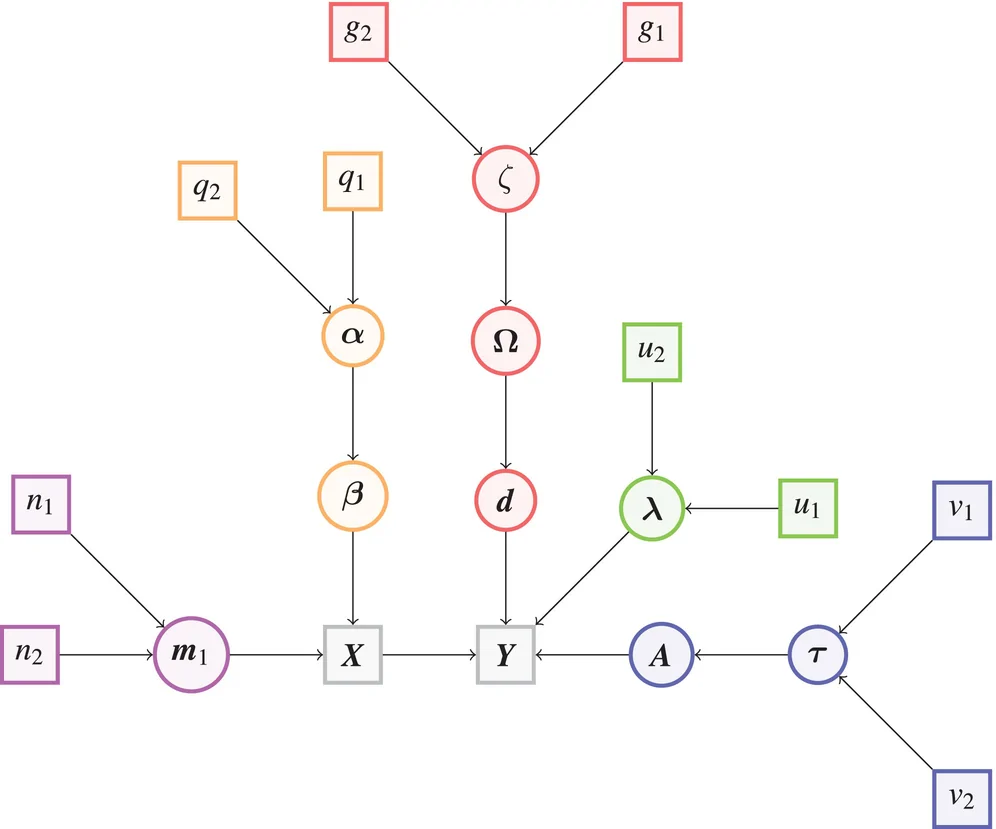
Graphical representation of the Bayesian model. Each node in a circle refers to a model parameter, nodes in squares are observables/hyperparameter. The link between two nodes represents a direct probabilistic dependence. The purple nodes ($m_{1},n_{1},n_{2}$) correspond to the HRF parameters and their hyperparameters, the orange nodes ($\alpha ,\beta ,q_{1},q_{2}$) indicate the activation coefficients and their hyperparameters, the red nodes ($\zeta ,\Omega ,d,g_{1},g_{2}$) show the parameters and hyperparameters related to the connectivity part, the green nodes ($\lambda ,u_{1},u_{2}$) correspond to the precision parameter of the error term, and the blue nodes ($A,\tau ,v_{1},v_{2}$) correspond to the AR part of the model.

We place a Gamma prior on ζ, such that $\zeta ∼𝒢(g_{1},g_{2})$, with the shape parameter $g_{1}=1$ and the rate parameter $g_{2}=0.1$. These values lead to a weakly informative prior, corresponding to $𝔼[\zeta ]=g_{1}/g_{2}=10$ and $\mathrm{Var}(\zeta )=g_{1}/g_{2}^{2}=100$. This choice follows Wang [36]: the large prior variance makes the prior diffuse enough that the posterior of ζ is driven by the data, while the finite mean keeps the induced Graphical Lasso penalty proper and the sampler numerically stable. Posterior inference is robust to moderate changes in $\left(g_{1},g_{2}\right)$, in the sensitivity checks of Wang [36]. The graphical representation of the parameters and hyperparameters of the Bayesian model is shown in Figure 2.

### 2.5 Posterior Computation

The model framework and prior structure allow sampling from the posterior distribution using the MCMC algorithm which is outlined in the Supporting Information (Appendix A). We have the full conditional distributions for parameters and hyperparameters of β, a, λ, α and τ, leading to a Gibbs sampling approach. The choice of conditionally conjugate priors for the model parameters largely simplifies the structure of the MCMC algorithm for posterior inference. For sampling the HRF parameters, we do not have a closed‐form conditional distribution, so we instead used a random walk Metropolis‐Hastings algorithm. We ran the MCMC algorithm for a total of 7000 iterations, with a burn‐in period of 3000 iterations. The posterior distributions of the unknown quantities of interest were approximated using the empirical distributions from the post‐burn‐in samples. To calibrate the acceptance ratio during burn‐in, we set the initial step size to 0.6 for all regions and subjects. The acceptance ratio was evaluated every 50 iterations in the 3000 burn‐in steps (i.e., 60 times). If the acceptance ratio was below 0.2 or above 0.5, the step size was adjusted by multiplying it by 1.3 or 0.7, respectively. These values were determined empirically by checking the MCMC diagnostics. This adjustment was only applied during the burn‐in, after which the step size remained fixed. This tuning approach helped keep the acceptance ratio within the desired range of 0.2 to 0.5. The sampling scheme is shown in Algorithm 1.

ALGORITHM 1 **MCMC sampling procedure for the proposed model.**

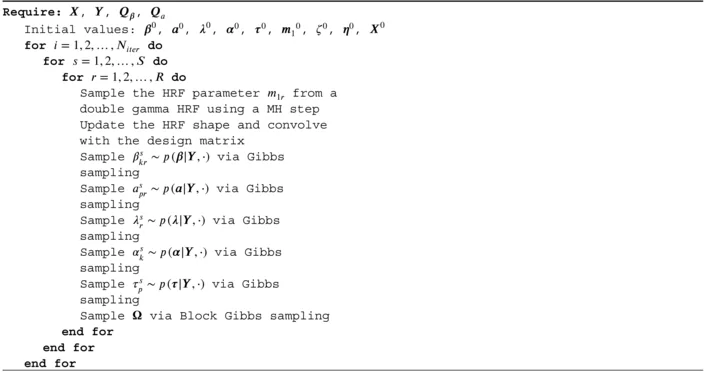

**FIGURE 3:**
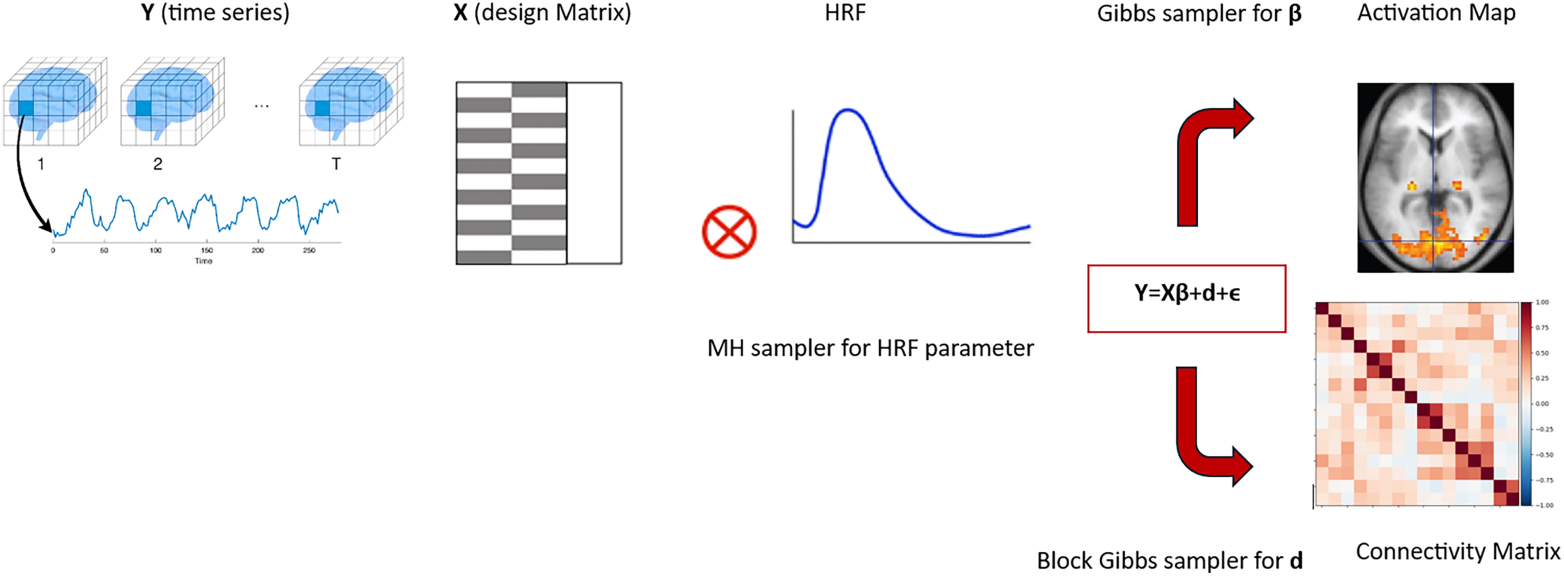
A general illustration of the proposed Bayesian model to jointly estimate brain activation and connectivity. The observed fMRI time series Y is modeled using a design matrix X convolved with HRF, whose parameters are sampled using a Metropolis‐Hastings (MH) algorithm. The linear model Y=Xβ+d+ϵ is estimated via Gibbs sampling for the activation coefficients β and block Gibbs sampling for the random effects d.^1^

To obtain a more interpretable measure of connectivity between brain regions, we examine the partial correlations. The partial correlation represents the relationship between two regions while controlling for the influence of all other regions [39], making it an appropriate metric to evaluate pairwise connections. Hence, for each posterior draw of the precision matrix Ω, we computed the corresponding partial correlation matrix using the prec2part function from the DensParcorr package in R [40]. The general illustration of the proposed model and the estimation procedure is shown in Figure 3.

## 3 Simulation Studies

To validate our proposed Bayesian model, we simulated synthetic data with a structure similar to that of real fMRI data. We evaluated the performance of the model in three different simulation studies to compare the characteristics of the posterior distributions obtained from MCMC sampling. We also compared the performance of our model to that of the standard GLM. The MCMC algorithm code is written and implemented in R version 4.5.0. To speed up the computations, we implemented the functions for the convolution of the design matrix with the HRF and for the likelihood calculation in Fortran 77.

We simulated 15 data sets each with a sample size of S=25 subjects, R=5 ROIs, with the number of time points per subject being fixed at T=214, and with an autoregressive order of P=2. The regressor $x_{tr,\cdot }^{s}=x_{tr,\cdot }$ was set to be the same for all subjects. A block experimental design was used to generate the regressors, consisting of alternating periods of stimulus presentation and rest. To construct the design matrix, we employed the specifydesign function from the neuRosim package in R [42]. The total duration of the simulated time series was set to 214 s, with a repetition time (TR) of 1 second. Each stimulus block lasted for 15 s. The design matrix was created using three different stimuli (features). While the experimental design was held constant across subjects, inter‐regional variability in the hemodynamic response was included by specifying region‐specific parameters for the HRF. In particular, we used the double gamma HRF, and varied the delay of response parameter ($m_{1}$) across regions, while within each region the HRF is shared across subjects, and we kept the effect size fixed at 1. Extending the model to subject‐ and region‐specific HRFs is straightforward in principle (it requires adding a subject index to $m_{1}$) but was deferred to future work for computational reasons; see Section 6. This allows the BOLD response to realistically vary by brain region. The resulting stimulus time series were convolved with their corresponding HRFs to obtain the design matrices used in the analysis. This convolution accounts for the expected delay between a stimulus and its associated BOLD response, as commonly modeled in fMRI studies [33]. The true values of the parameters β,a, and $z_{\cdot ,r}^{s}$ are simulated based on the model assumptions and fixed values of $\alpha_{1}^{s}=100$, $\alpha_{2}^{s}=100$, $\alpha_{3}^{s}=200$, and $\tau_{1}^{s}=\tau_{2}^{s}=1000$. To define the observation‐level precision for each subject and region, we first computed the variance of the signal generated by the design matrix and the corresponding activation coefficients. Specifically, for each subject s and region r, we calculated the variance of the signal as $\mathrm{Var}\left[{\left(x_{tr,\cdot }^{s}\right)}^{\prime }\beta_{\cdot ,r}^{s}\right]$, where $x_{tr,\cdot }^{s}$ denotes the design matrix and $\beta_{\cdot ,r}^{s}$ the vector of activation effects. The precision $\lambda_{r}^{s}$ was then defined as:

$$\frac{1}{\lambda_{r}^{s}}=\frac{\mathrm{Var}\left[{\left(x_{tr,\cdot }^{s}\right)}^{\prime }\beta_{\cdot ,r}^{s}\right]}{\text{SNR}},$$

where SNR is the signal‐to‐noise ratio. This formulation allows for subject‐ and region‐specific noise levels in the simulated data. We assessed the model under different signal to noise ratios (SNRs): high (SNR=10), moderate (SNR=3) and low (SNR=0.5). These values are in line with commonly used definitions and empirical ranges discussed by Welvaert and Rosseel [43], and allow us to evaluate model performance under both favorable and challenging noise regimes, particularly in low‐SNR settings where classical GLM approaches are known to struggle.

**FIGURE C1:**
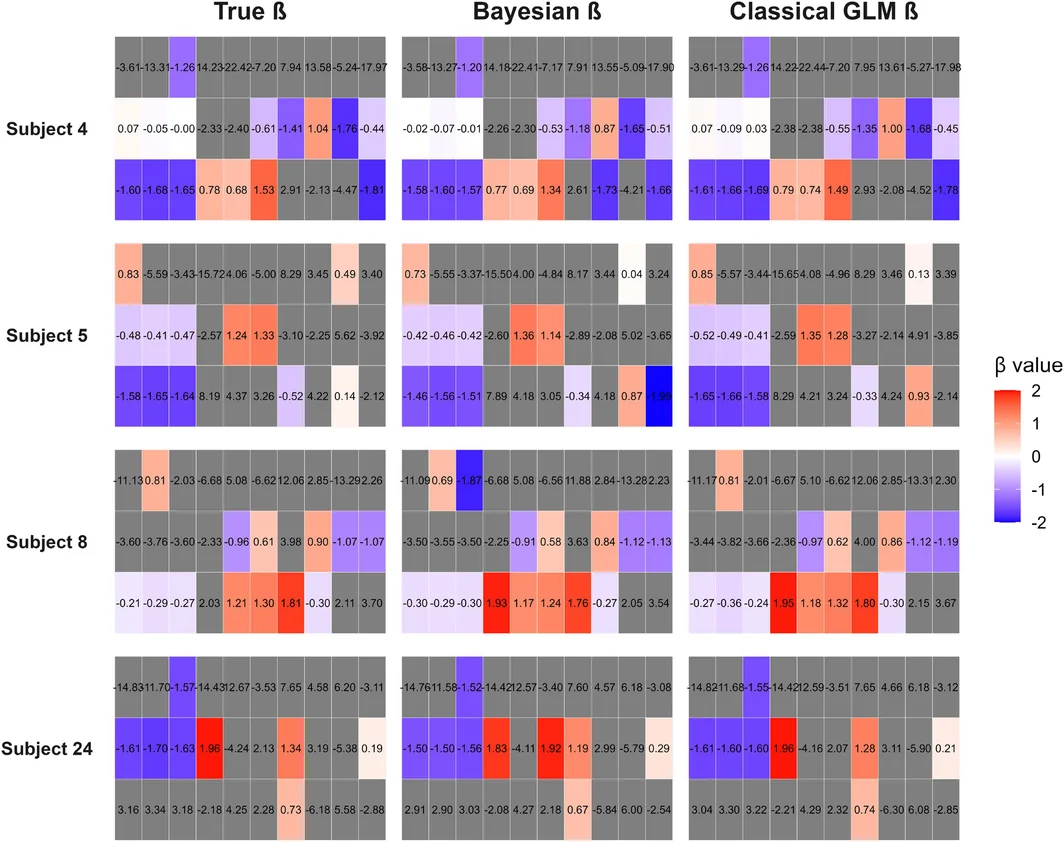
Experiment on simulated data for SNR = 10, results on (β) activation coefficient estimation: true activation maps (left), posterior estimated maps estimated via MCMC (middle) and maps estimated with classical GLM (right). Results are shown for four subjects, three stimuli (heatmap rows), and ten ROIs (heatmap columns).

**FIGURE C2:**
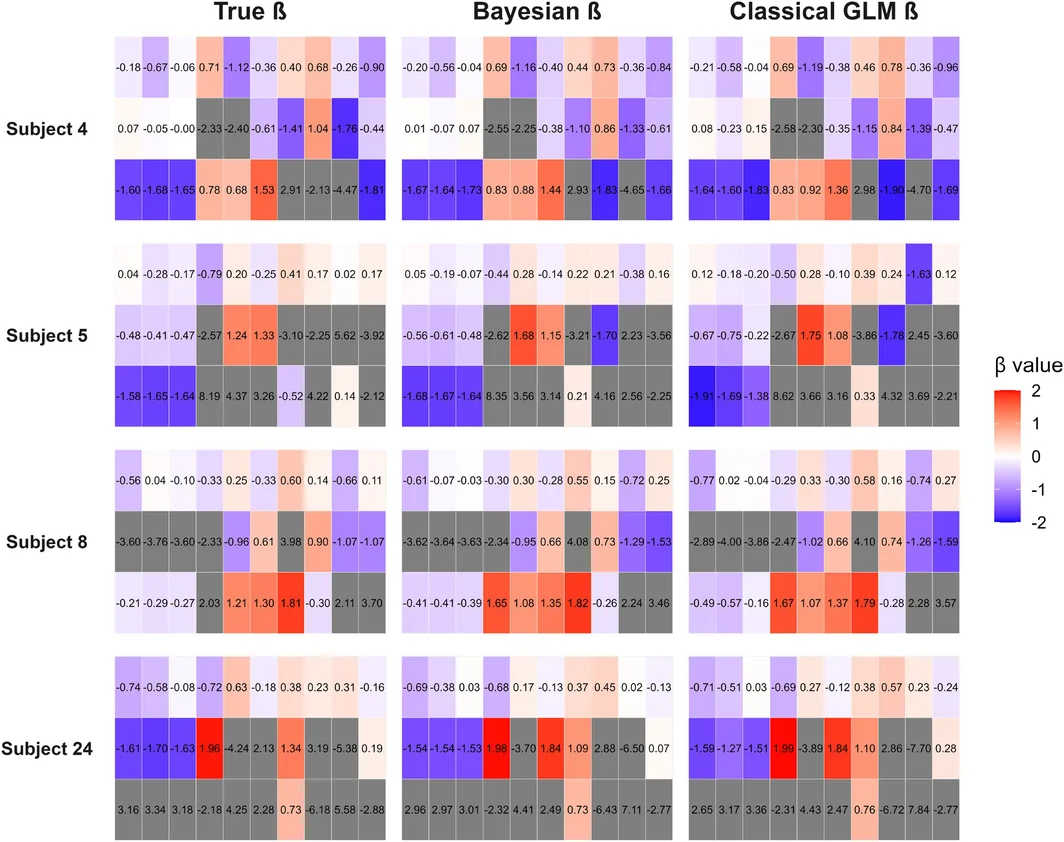
Experiment on simulated data for SNR = 0.5, results on (β) activation coefficient estimation: true activation maps (left), posterior estimated maps estimated via MCMC (middle) and maps estimated with classical GLM (right). Results are shown for four subjects, three stimuli (heatmap rows), and ten ROIs (heatmap columns).

**FIGURE C3:**
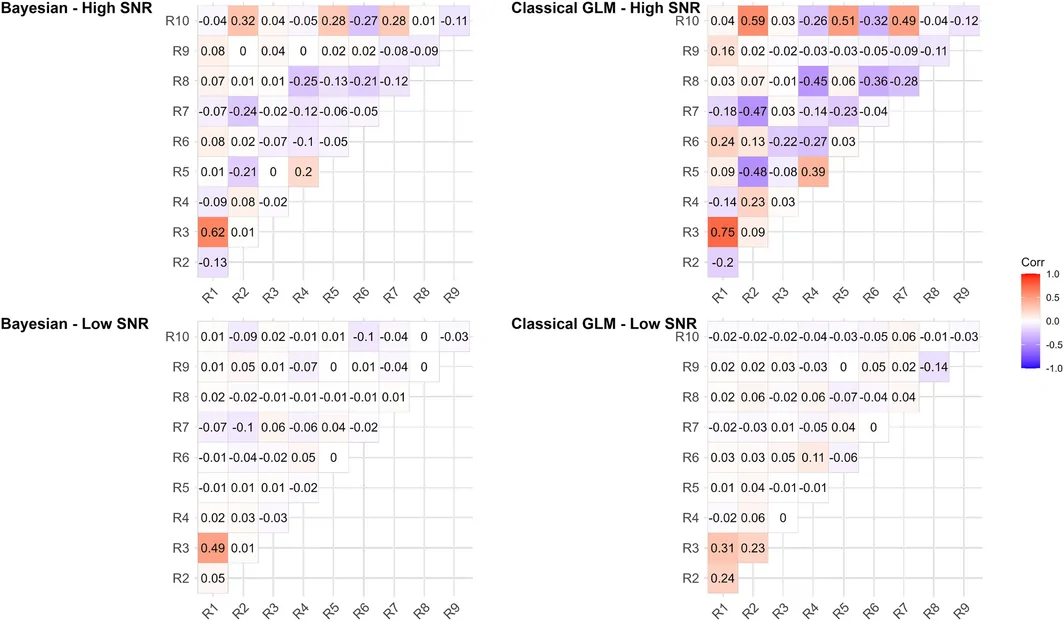
Estimates of the partial correlation for all possible region pairs using the Bayesian model and classical GLM under SNR = 10 (high) and SNR = 0.5 (low). The true partial correlation value for region pair (R1,R3) is set to 0.7 and the rest of the partial correlations are set to zero.

**FIGURE C4:**
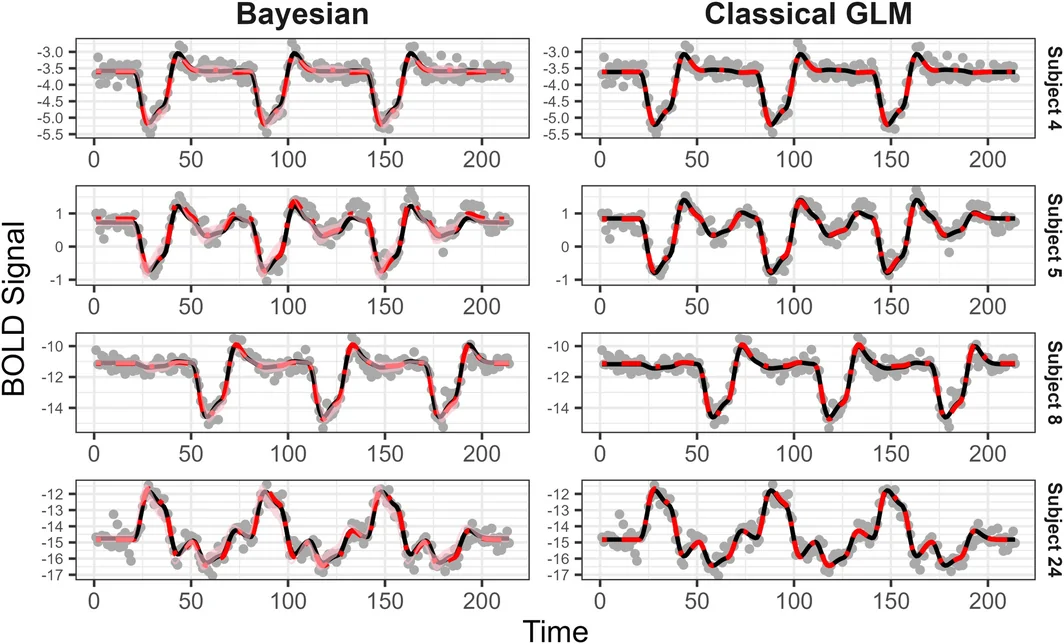
Model fit comparison for SNR = 10. The left column shows the model fit from Bayesian model and the right column shows the model fit from the classical GLM. The dots in gray indicate the simulated response, the black solid line is the estimated response from the model, the red dash‐dot line indicates estimated response for true values of parameters from simulation study, and the pink shadow area represents the 95% credible interval.

**FIGURE C5:**
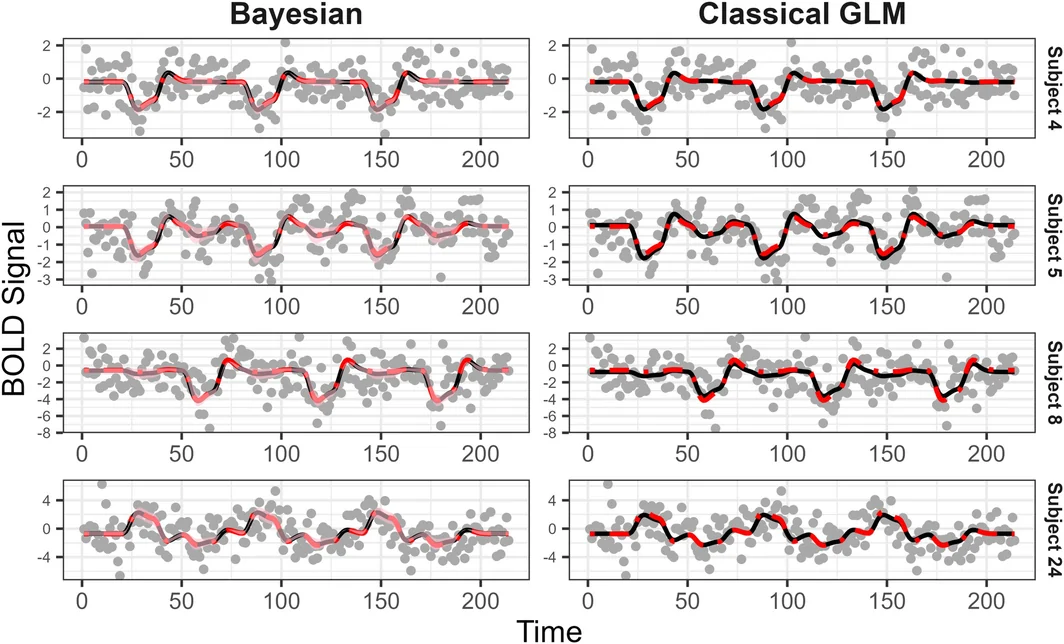
Model fit comparison for SNR = 0.5. The left column shows the model fit from Bayesian model and the right column shows the model fit from the classical GLM. The dots in gray indicate the simulated response, the black solid line is the estimated response from the model, the red dash‐dot line indicates estimated response for true values of parameters from simulation study, and the pink shadow area represents the 95% credible interval.

**FIGURE C6:**
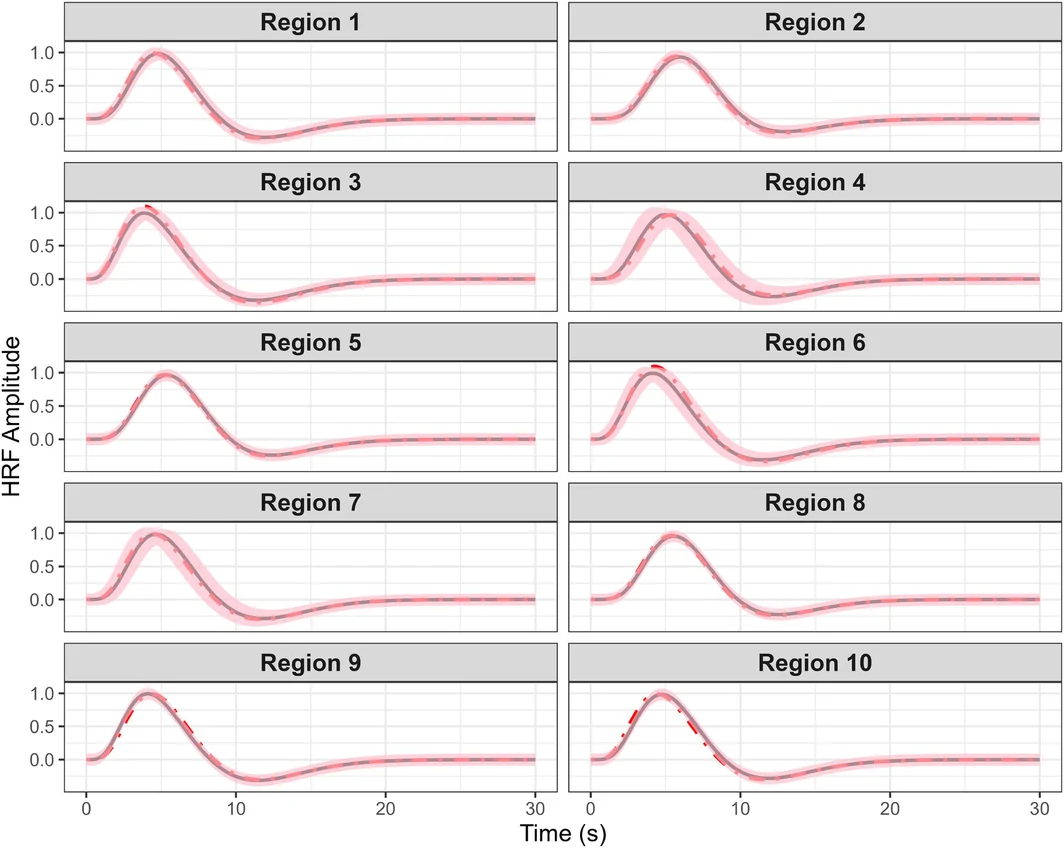
Posterior HRF results for ten regions and subject 24 under SNR = 10 (high). The black solid line represents the estimated posterior median HRF, the red dash‐dot line indicates the simulated HRF, and the pink shadow area represents the 95% credible interval.

**FIGURE C7:**
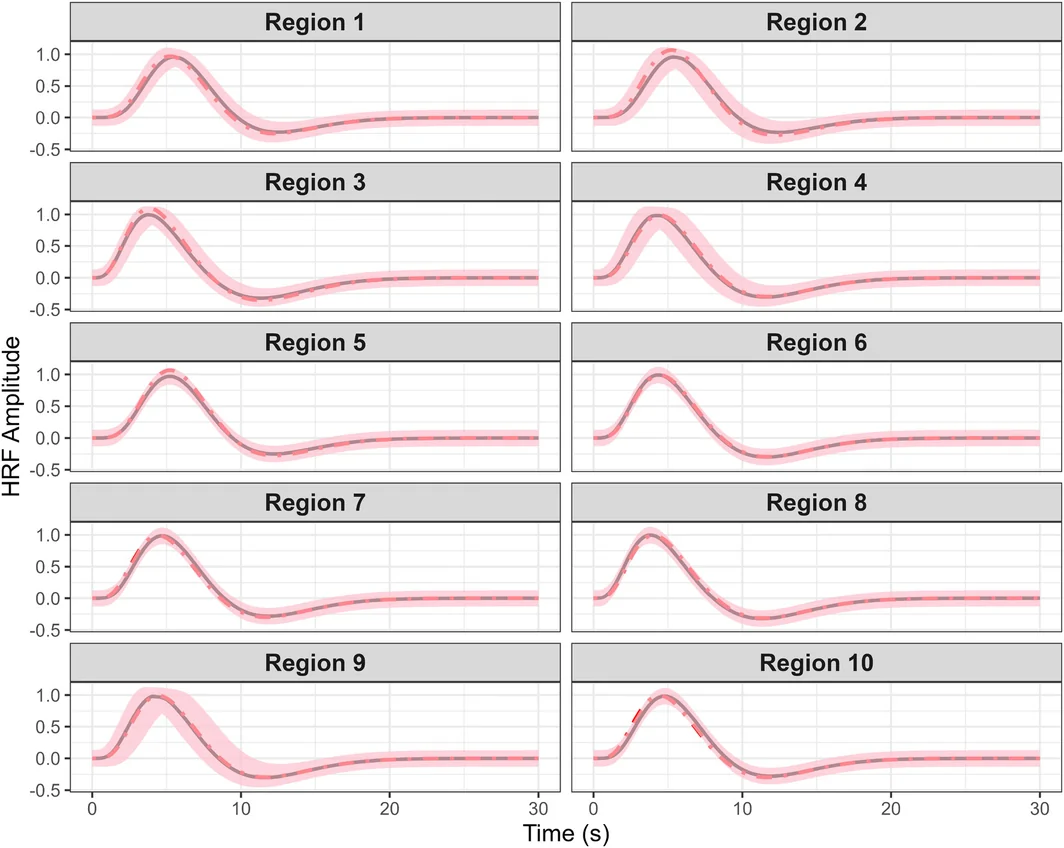
Posterior HRF results for ten regions and subject 24 under SNR = 0.5 (low). The black solid line represents the estimated posterior median HRF, the red dash‐dot line indicates the simulated HRF, and the pink shadow area represents the 95% credible interval.

The connectivity between regions was simulated by setting two pairs of the five regions (regions 1 and 3) to have a region‐wide correlation of 0.7, while all other regions were assigned correlations of zero. A covariance matrix ($\Omega^{-1}$) was created from this correlation matrix, and the region effects for subject s were simulated from a multivariate normal distribution with mean zero and covariance $\Omega^{-1}$, $d^{s}∼𝒩(0,\Omega^{-1})$. Given the parameter values, the data Y were simulated from the model in Equation (4). We also evaluated the model by increasing the number of ROIs from 5 to 10 for both high‐and‐low SNRs regimes. The results indicated that the model remained stable under this higher‐dimensional configuration. The results are shown in Appendix C (Figures C1, C2, C3, C4, C5, C6, C7). To assess the accuracy of the model with respect to point estimation, we use the simulation replicates and the known true values of the model parameters to estimate the average squared bias (ASBIAS) and the average mean squared error (AMSE) of estimators based on the posterior median, where the average is taken across regions. Also we use the average marginal variance (AVAR). Letting ${\hat{\beta }}_{krj}^{s}$ denote the posterior median estimate of $\beta_{kr}^{s}$ obtained from the $j^{th}$ (j=1,…,J) simulation replicate, and $\sigma^{2}({\hat{\beta }}_{krj}^{s})$ denote the corresponding posterior variance, the three measures above for $\beta_{k,\cdot }^{s}$ (where k corresponds to the $k^{th}$ regressor for subject s) are computed as:

(12) $$\begin{array}{ll} \text{ASBIAS}\left(\beta_{k,\cdot }^{s}\right) & =\frac{1}{R}\overset{R}{\underset{r=1}{\sum }}{\left(\overset{J}{\underset{j=1}{\sum }}\frac{{\hat{\beta }}_{krj}^{s}}{J}-\overset{J}{\underset{j=1}{\sum }}\frac{\beta_{krj}^{s}}{J}\right)}^{2}, \end{array}$$

(13) $$\begin{array}{ll} \text{AMSE}\left(\beta_{k,\cdot }^{s}\right) & =\frac{1}{RJ}\overset{R}{\underset{r=1}{\sum }}\overset{J}{\underset{j=1}{\sum }}{\left({\hat{\beta }}_{krj}^{s}-\beta_{krj}^{s}\right)}^{2}, \end{array}$$

(14) $$\begin{array}{ll} \text{AVAR}\left(\beta_{k,\cdot }^{s}\right) & =\frac{1}{RJ}\overset{R}{\underset{r=1}{\sum }}\overset{J}{\underset{j=1}{\sum }}\sigma^{2}\left({\hat{\beta }}_{krj}^{s}\right). \end{array}$$

We computed these measures for each subject. The same measures are applied to the autoregressive coefficients $a_{p,\cdot }^{s}$. The summary statistics are computed and their values are listed in Table 1 in the next section. To capture the uncertainty in the posterior estimates, we computed their 95% credible interval (CI).

**TABLE 1:** Performance metrics (ASBIAS, AMSE, AVAR) for Bayesian and classical GLM under varying SNR levels.

SNR	Metric	Param	Bayesian				classical GLM			
			s=4	s=5	s=8	s=24	s=4	s=5	s=8	s=24
10	ASBIAS	$\beta_{1}$	0.005	0.002	0.003	0.0009	0.001	0.0001	0.0001	0.0001
		$\beta_{2}$	0.0009	0.003	0.002	0.002	0.001	0.001	0.001	0.0005
		$\beta_{3}$	0.03	0.15	0.008	0.002	0.001	0.15	0.0003	0.0004
		$a_{1}$	0.002	0.001	0.0008	0.0003	—	—	—	—
		$a_{2}$	0.0008	0.0007	0.0006	0.002	—	—	—	—
10	AMSE	$\beta_{1}$	0.08	0.04	0.07	0.04	0.01	0.01	0.004	0.004
		$\beta_{2}$	0.02	0.04	0.04	0.09	0.006	0.008	0.008	0.006
		$\beta_{3}$	0.02	0.01	0.02	0.01	0.001	0.005	0.001	0.001
		$a_{1}$	0.003	0.002	0.001	0.002	—	—	—	—
		$a_{2}$	0.001	0.002	0.002	0.004	—	—	—	—
10	AVAR	$\beta_{1}$	0.03	0.02	0.02	0.03	—	—	—	—
		$\beta_{2}$	0.03	0.02	0.02	0.03	—	—	—	—
		$\beta_{3}$	0.01	0.03	0.01	0.03	—	—	—	—
		$a_{1}$	0.002	0.002	0.002	0.002	—	—	—	—
		$a_{2}$	0.002	0.001	0.002	0.002	—	—	—	—
0.5	ASBIAS	$\beta_{1}$	0.01	0.01	0.002	0.0008	0.01	0.01	0.004	0.01
		$\beta_{2}$	0.01	0.01	0.01	0.01	0.01	0.02	0.01	0.01
		$\beta_{3}$	0.007	0.001	0.002	0.002	0.01	0.005	0.002	0.003
		$a_{1}$	0.0004	0.0001	0.0001	0.0003	—	—	—	—
		$a_{2}$	0.0001	0.0002	0.0004	0.0001	—	—	—	—
0.5	AMSE	$\beta_{1}$	0.14	0.09	0.06	0.04	0.22	0.14	0.07	0.08
		$\beta_{2}$	0.05	0.14	0.10	0.13	0.16	0.23	0.16	0.14
		$\beta_{3}$	0.03	0.01	0.01	0.02	0.11	0.02	0.01	0.02
		$a_{1}$	0.004	0.002	0.004	0.005	—	—	—	—
		$a_{2}$	0.003	0.002	0.004	0.002	—	—	—	—
0.5	AVAR	$\beta_{1}$	0.15	0.10	0.09	0.09	—	—	—	—
		$\beta_{2}$	0.14	0.10	0.08	0.09	—	—	—	—
		$\beta_{3}$	0.02	0.01	0.01	0.01	—	—	—	—
		$a_{1}$	0.002	0.002	0.002	0.002	—	—	—	—
		$a_{2}$	0.002	0.002	0.002	0.002	—	—	—	—

In addition, to quantify uncertainty in the estimated partial correlations, we computed Bayesian credible intervals for each pair of regions. Posterior samples of the precision matrix were first obtained and transformed into partial correlations at each iteration. Credible intervals were computed as the 2.5% and 97.5% quantiles of the resulting posterior distribution. All analyses were repeated under high (SNR = 10) and low (SNR = 0.5) SNRs, and results were aggregated across simulation replicates.

## 4 Results

**FIGURE 4:**
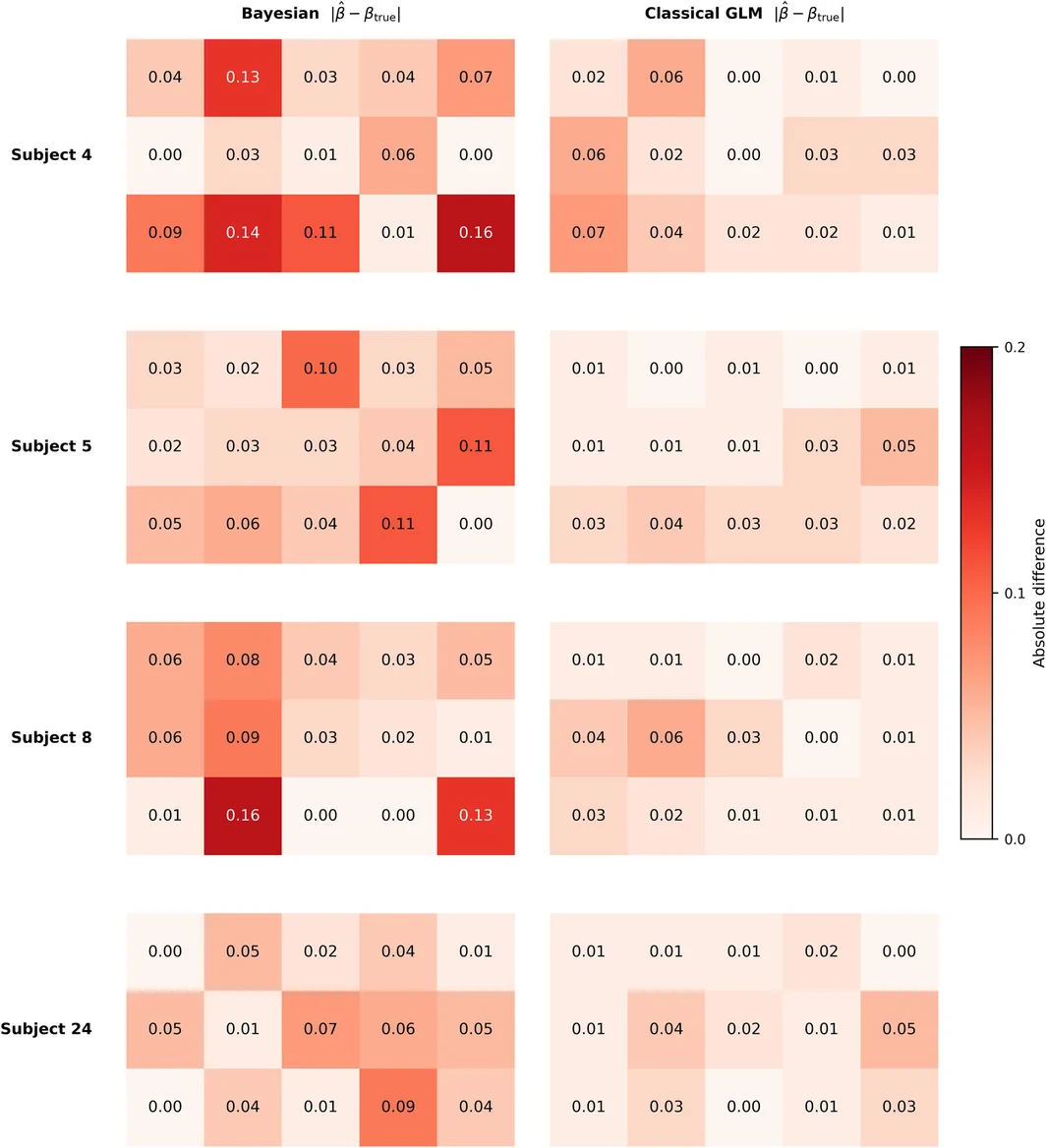
Experiment on simulated data for SNR =10, absolute error of activation coefficient (β) estimates: heatmaps show the element‐wise absolute difference $\left|\hat{\beta }-\beta_{\text{true}}\right|$ between estimated and true activation maps for the Bayesian posterior estimate obtained via MCMC (left) and the classical GLM estimate (right). Results are shown for four subjects, three stimuli (heatmap rows), and five ROIs (heatmap columns). Darker cells indicate larger deviations from the ground truth; the color bar shows the absolute difference.

**FIGURE 5:**
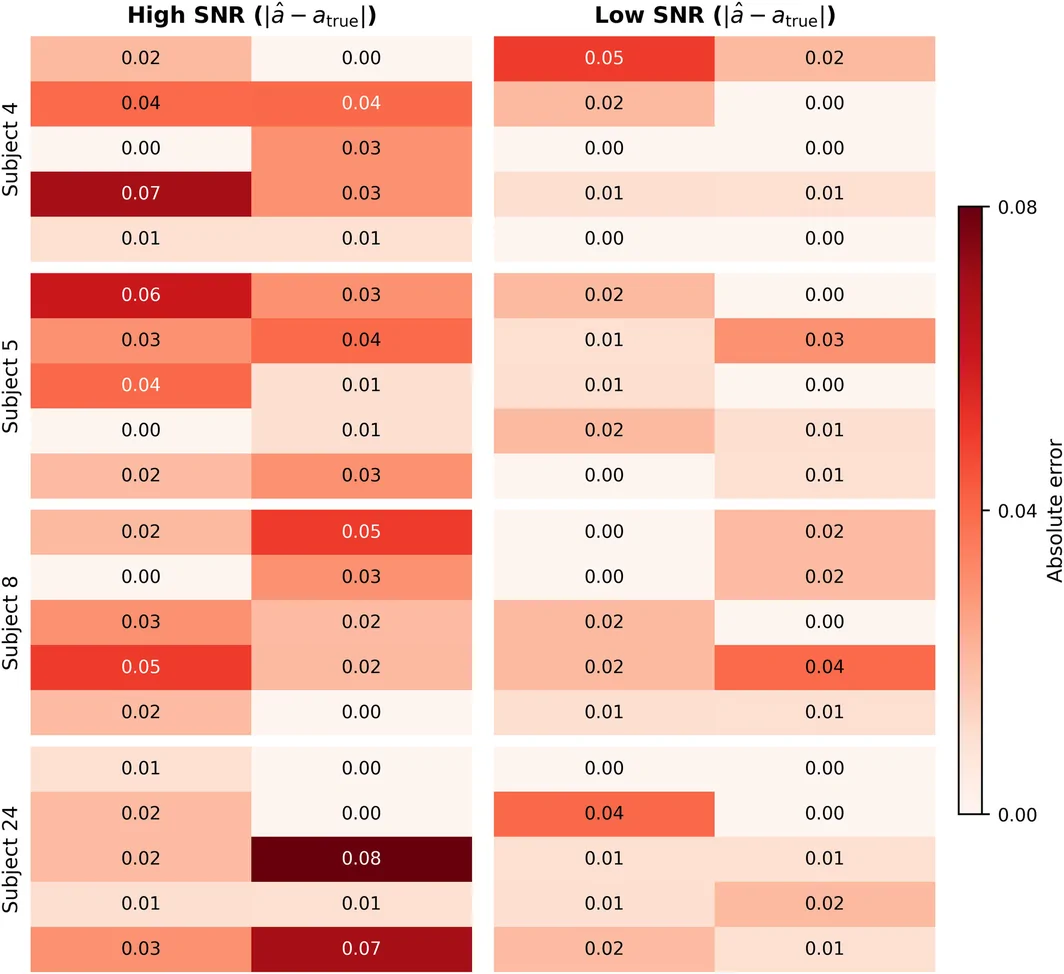
Experiment on simulated data comparing high and low signal‐to‐noise ratio (SNR) settings. The figure shows element‐wise absolute error of autoregressive coefficient estimates, computed as $\left|\hat{a}-a_{\text{true}}\right|$. Heatmaps display results for four subjects (rows) and two SNR conditions (columns): high SNR (left) and low SNR (right), with autoregressive orders two and five ROIs. Each cell represents the absolute deviation between Bayesian posterior median estimates obtained via MCMC and the corresponding true coefficients. Darker colors indicate larger estimation errors, and the shared color bar represents the magnitude of absolute error across all panels.

**FIGURE 6:**
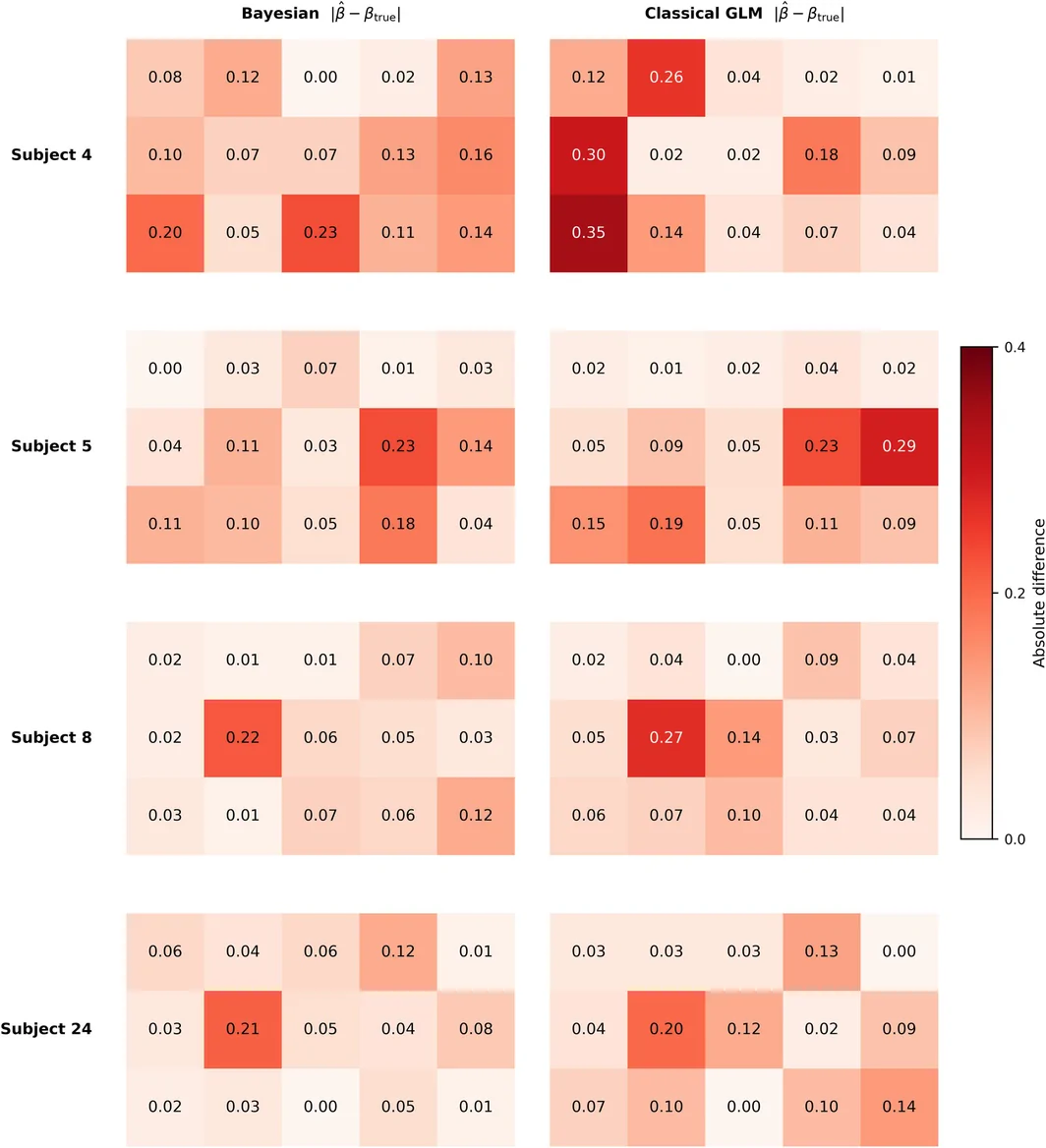
Experiment on simulated data for SNR =0.5, absolute error of activation coefficient (β) estimates: heatmaps show the element‐wise absolute difference $\left|\hat{\beta }-\beta_{\text{true}}\right|$ between estimated and true activation maps for the Bayesian posterior estimate obtained via MCMC (left) and the classical GLM estimate (right). Results are shown for four subjects, three stimuli (heatmap rows), and five ROIs (heatmap columns). Darker cells indicate larger deviations from the ground truth; the color bar shows the absolute difference.

**FIGURE B1:**
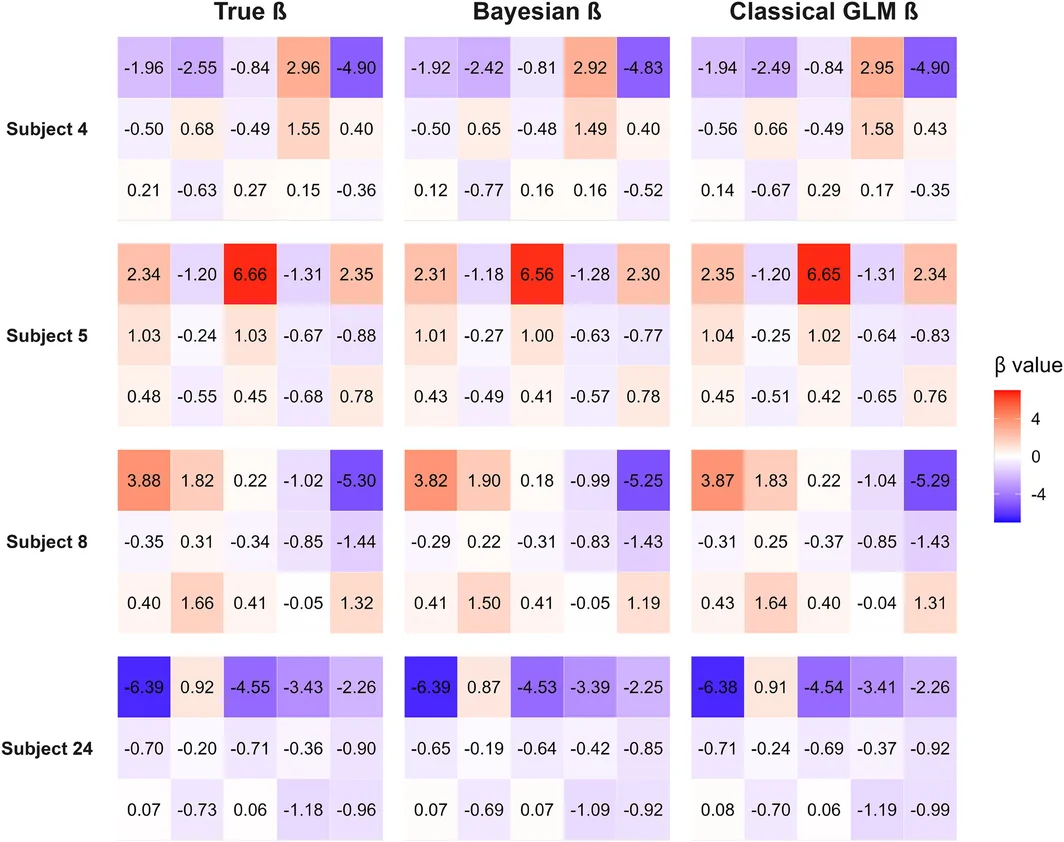
Experiment on simulated data for SNR = 10, results on (β) activation coefficient estimation: true activation maps (left), posterior estimated maps estimated via MCMC (middle) and maps estimated with classical GLM (right). Results are shown for four subjects, three stimuli (heatmap rows), and five ROIs (heatmap columns).

**FIGURE B2:**
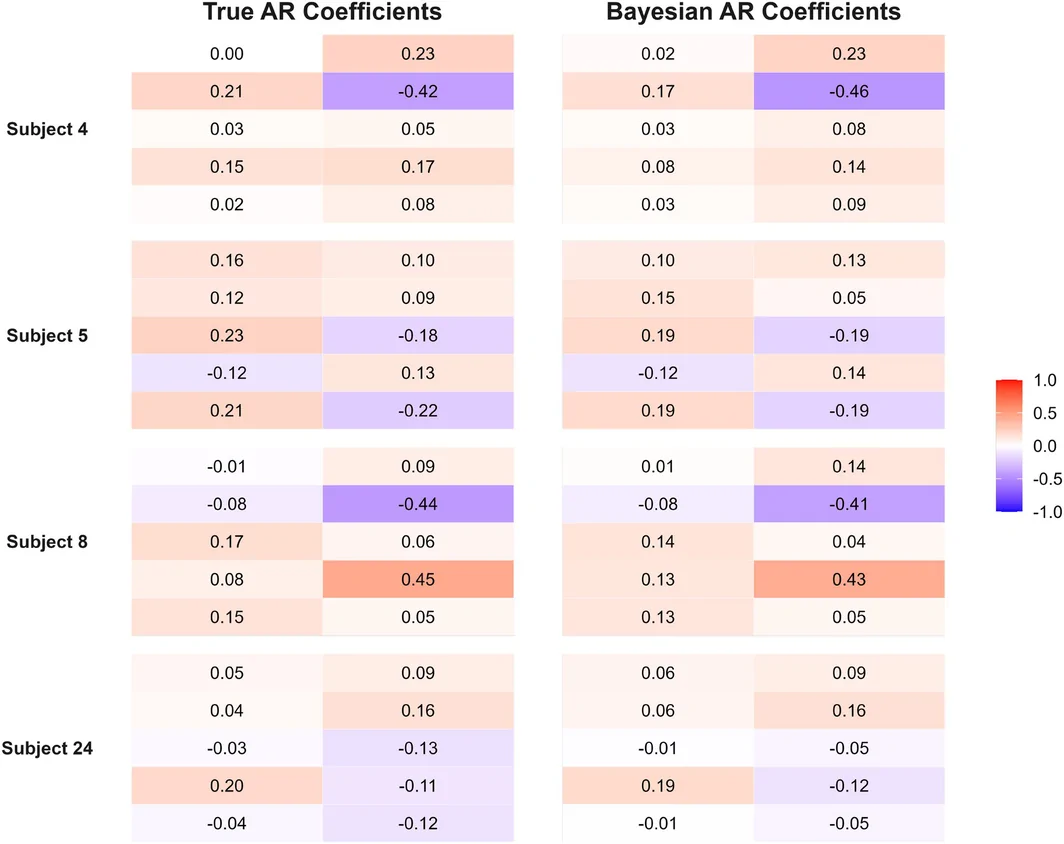
Experiment on simulated data for SNR = 10, results on (a) autoregressive coefficient estimation: true autoregressive coefficients (left), estimated autoregressive coefficients from Bayesian model (right). Results are shown for four subjects, the autoregressive order of two (heatmap columns), and five ROIs (heatmap rows).

**FIGURE B3:**
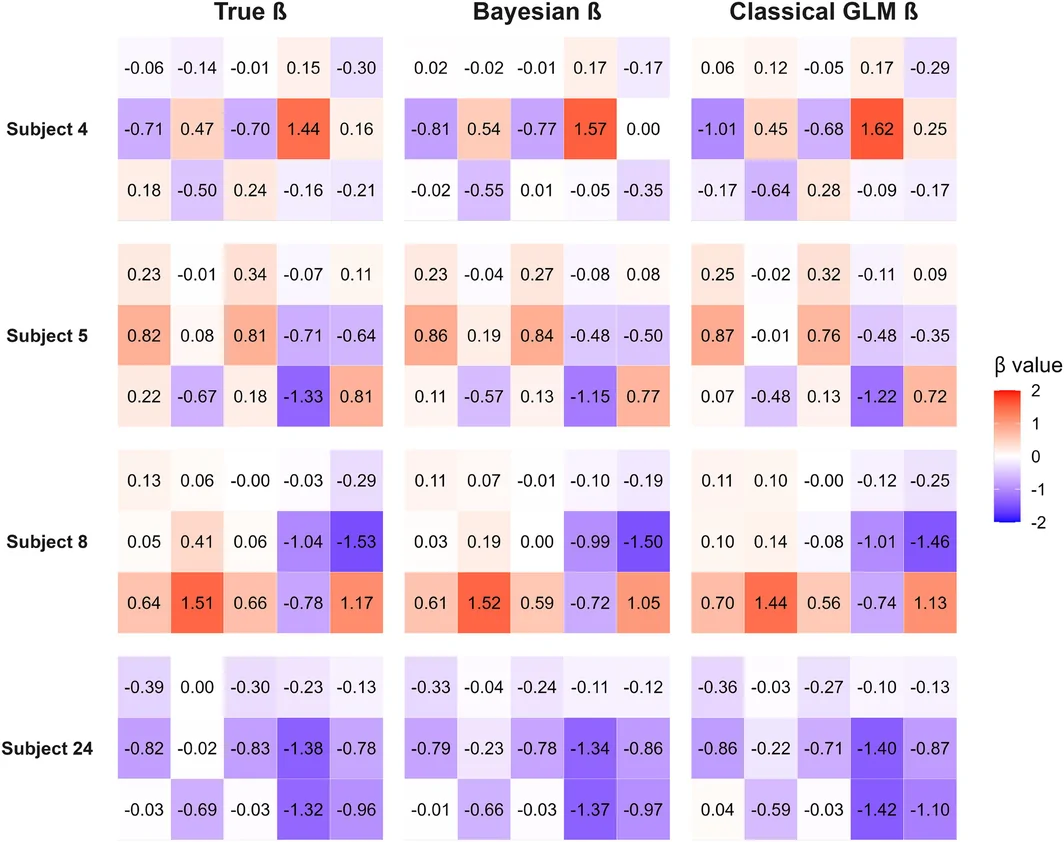
Experiment on simulated data for SNR = 0.5, results on β activation coefficient estimation: true activation maps (left), posterior estimated maps estimated via MCMC (middle) and maps estimated with classical GLM (right). Results are shown for four subjects, three stimuli (heatmap rows), and five ROIs (heatmap columns).

**FIGURE B4:**
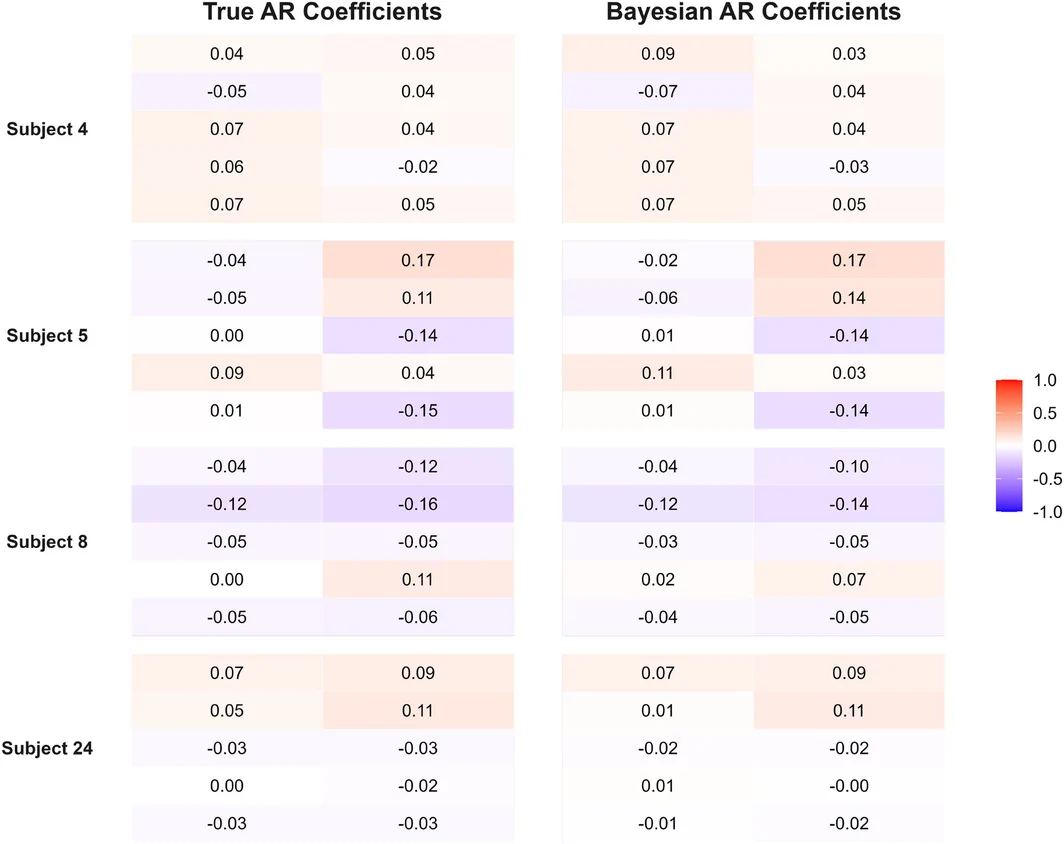
Experiment on simulated data for SNR = 0.5, results on (a) autoregressive coefficient estimation: true autoregressive coefficients (left), estimated autoregressive coefficients from Bayesian model (right). Results are shown for four subjects, the autoregressive order of two (heatmap columns), and five ROIs (heatmap rows).

The Bayesian and standard GLM methods are applied to the simulated datasets. In the first simulation study, we aim to assess the performance of our model under high SNR(=10). Figure 4 displays heatmaps of the absolute differences between the estimated and true values of $\beta_{kr}^{s}$ from both Bayesian method and classical GLM. The reported values are averaged across simulation replicates for both methods. Figure 5 shows the autoregressive parameters $a_{pr}^{\left(s\right)}$ under high and low SNR settings. The Bayesian MCMC estimates closely recover the true parameters in both regimes, with generally small absolute errors across subjects and coefficients. Differences between high‐ and low‐SNR conditions are modest and not uniformly directional across all entries. The posterior median estimates together with the ground‐truth values $\beta_{kr}^{s}$ for k∈{1,2,3} and r∈{1,2,3,4,5}, and $a_{pr}^{s}$ for p∈{1,2} in four selected subjects are provided in the Appendix (Figures B1 and B2). In this scenario, the results obtained from Bayesian and classical GLM are very similar and both correspond well to the ground truth. In the second simulation study, we aim to assess the performance of our model under low SNR(=0.5). The absolute differences between the estimated and ground‐truth of $\beta_{kr}^{s}$ from the Bayesian and classical GLM are shown in Figure 6, and $\beta_{kr}^{s}$ from the Bayesian and classical GLM are shown in Figure B3 in the Appendix. In our Bayesian model, the average posterior median estimates of $\beta_{kr}^{s}$ appear to be closer to the ground truth compared to the estimates from the classical GLM. The posterior medians of $a_{pr}^{\left(s\right)}$ together with their ground truth values are shown in Figure B4 in the Appendix. Also in this case, the estimations from the Bayesian model are aligned well with the ground truth.

In order to further assess the performance of our model, we repeated the simulations 15 times, and summary statistics (AMSE, ASBIAS, AVAR) are computed for $\beta_{k,\cdot }^{s}$ and $a_{p,\cdot }^{s}$. The results from the Bayesian and classical GLM for SNR=10 and SNR=0.5 are shown in Table 1. Since the classical GLM implementation does not provide the point estimates for the autoregressive coefficients, these cells are blank in the table. As reported in Table 1, for high SNR, the classical GLM seems to work slightly better compared to our model. This result is anticipated since, in high SNR scenarios, ordinary least squares (OLS) estimators are unbiased and have lower variance; however, the classical GLM does not provide uncertainty quantification. When decreasing the SNR to 0.5, our Bayesian model produces better estimates closer to the true values. In this scenario, ASBIAS and AMSE are lower for the Bayesian model. The results indicate that our Bayesian model outperforms the standard GLM when there is little data, which is typically the case in fMRI experiments. Generally, simulation studies with high SNR seem to produce a smaller squared bias in point estimation and a smaller AMSE for the classical GLM. However, the average marginal posterior variance (AVAR) indicates that our Bayesian model does not exhibit an overconfidence problem in high and low SNR settings.

**FIGURE 7:**
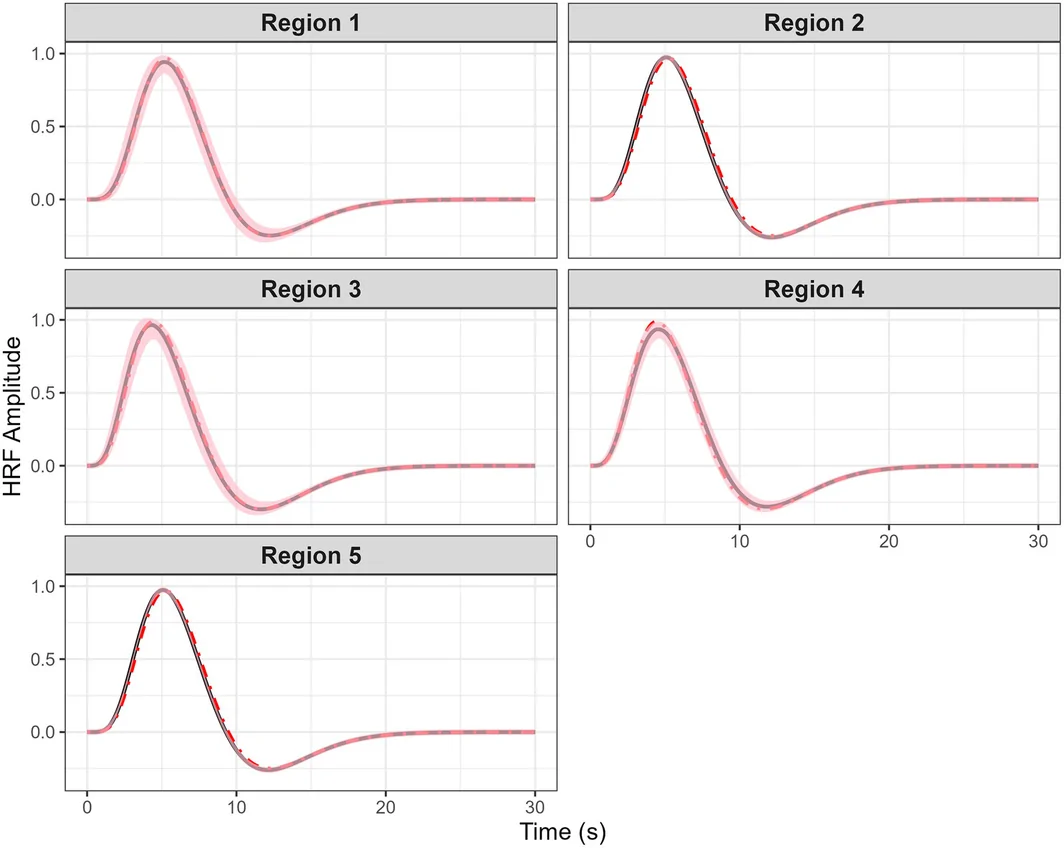
Posterior HRF results for five regions and subject 24 under SNR = 10 (high). The black solid line represents the estimated posterior median HRF, the red dash‐dot line indicates the simulated HRF, and the pink shadow area represents the 95% credible interval.

**FIGURE 8:**
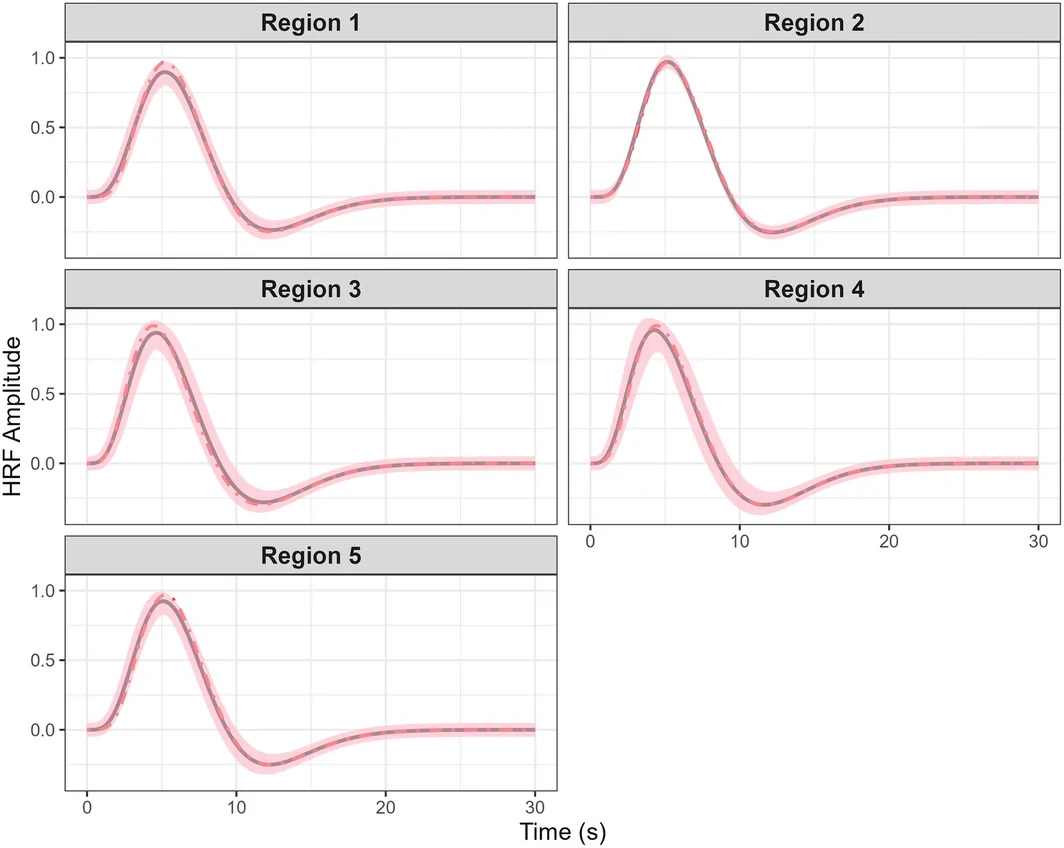
Posterior HRF results for five regions and subject 24 under SNR = 0.5 (low). The black solid line represents the estimated posterior median HRF, the red dash‐dot line indicates the simulated HRF, and the pink shadow area represents the 95% credible interval.

Posterior HRF estimates for five brain regions from Subject 24 under two SNR conditions are shown in Figures 7 and 8. Under high SNR (SNR=10, Figure 7), uncertainty varies by region. In Regions 2 and 5, the estimates match the ground truth almost perfectly, and the highly informative data likelihood drastically narrows the credible intervals. However, a slight peak mismatch occurs in Region 4, where the true response overshoots the estimate with a slightly wider credible interval. The increased variation across subjects in the high SNR setting explains this discrepancy and underscores the clear necessity of estimating HRF parameters at the subject level rather than relying solely on regional constraints. It also highlights the need for more flexible HRF estimation approaches, which are discussed further in Section 6.

Under low SNR (SNR=0.5, Figure 8), the estimated HRFs closely track the simulated ground truth across regions. In this high‐noise setting, the flat data likelihood allows the regional priors to dominate, which smooths out noise fluctuations. Nevertheless, under this condition, the model tends to underestimate the true peak height of the HRF in Regions 1, 3 and 5. Despite these minor variations in peak tracking and uncertainty, the general shape of HRF is well recovered under both noise conditions.

**FIGURE 9:**
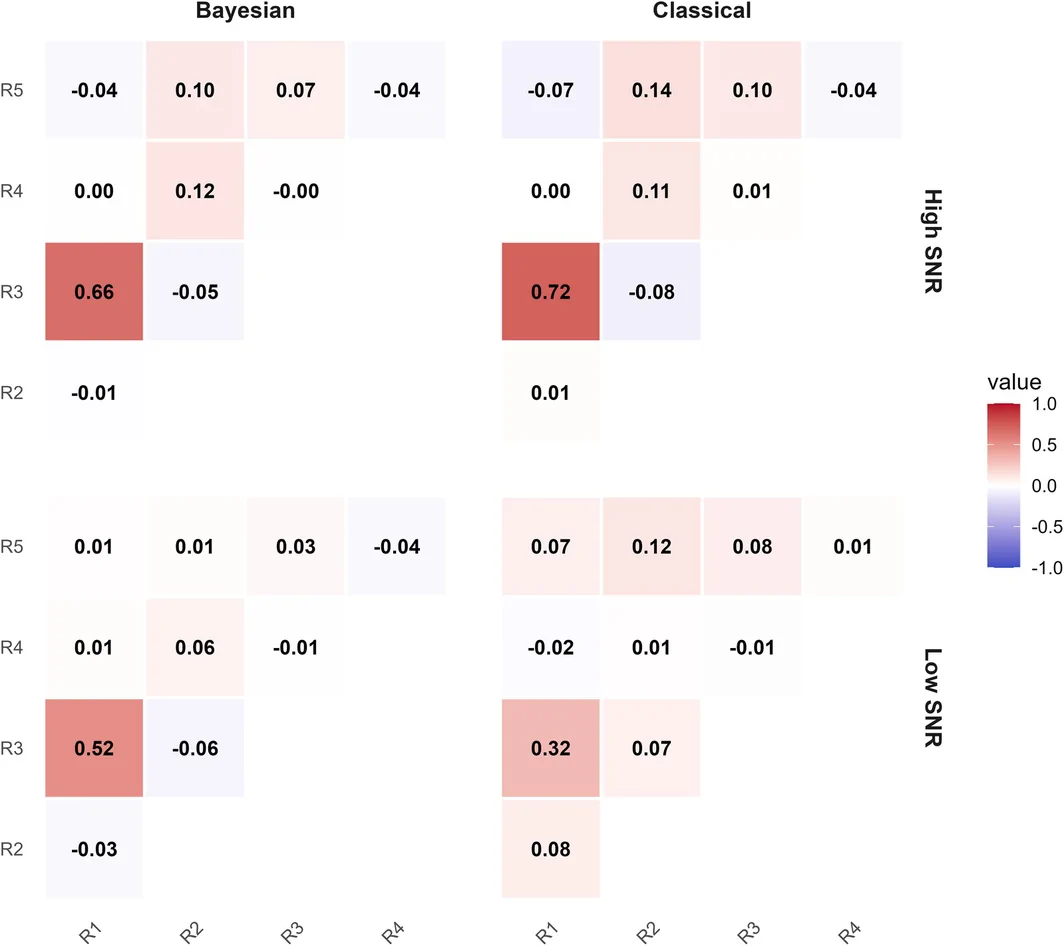
Estimates of the partial correlation for all possible region pairs using the Bayesian model and classical GLM under SNR = 10 (high) and SNR = 0.5 (low). The true partial correlation value for region pair R1,R3 is set to 0.7, and the rest of the partial correlations are set to zero.

**FIGURE B5:**
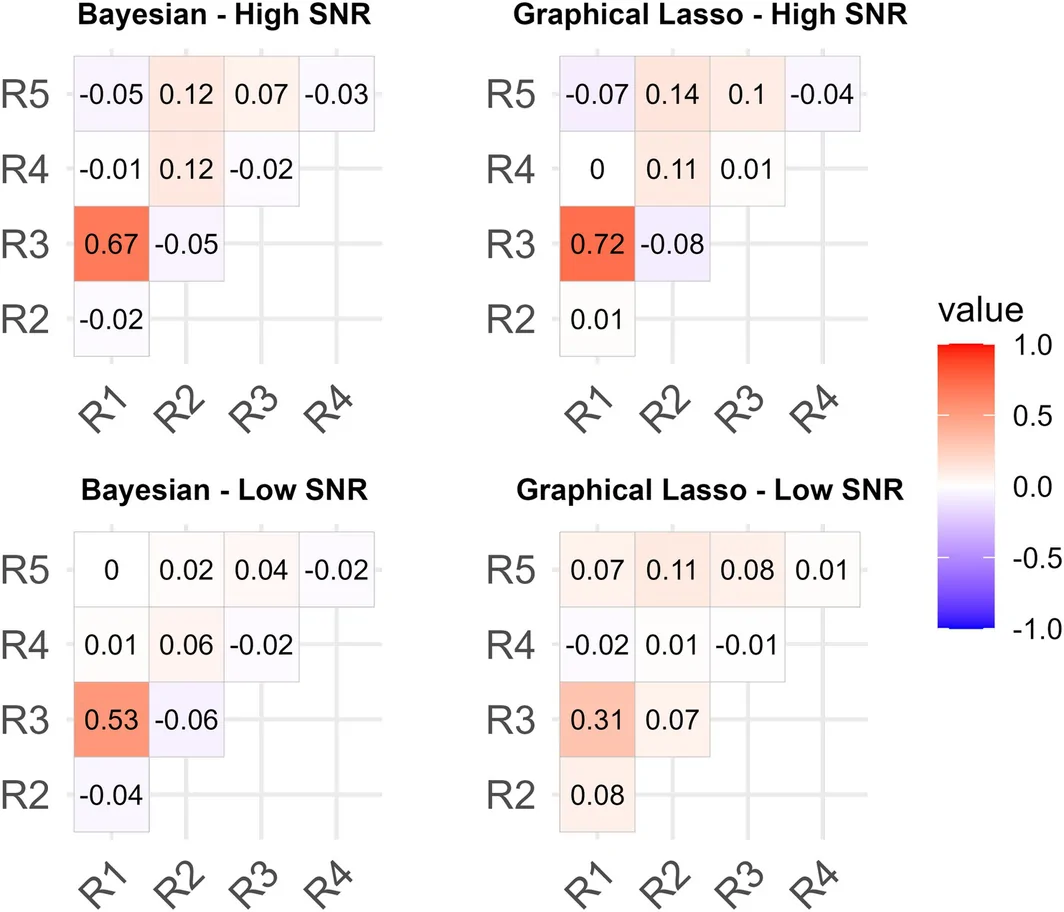
Estimates of the partial correlation for all possible region pairs using the Bayesian model and graphical lasso (regularization parameter = 0.01) under SNR = 10 (high) and SNR = 0.5 (low). The true partial correlation value for region pair (R1, R3) is set to 0.7 and the rest of the partial correlations are set to zero.

**FIGURE B6:**
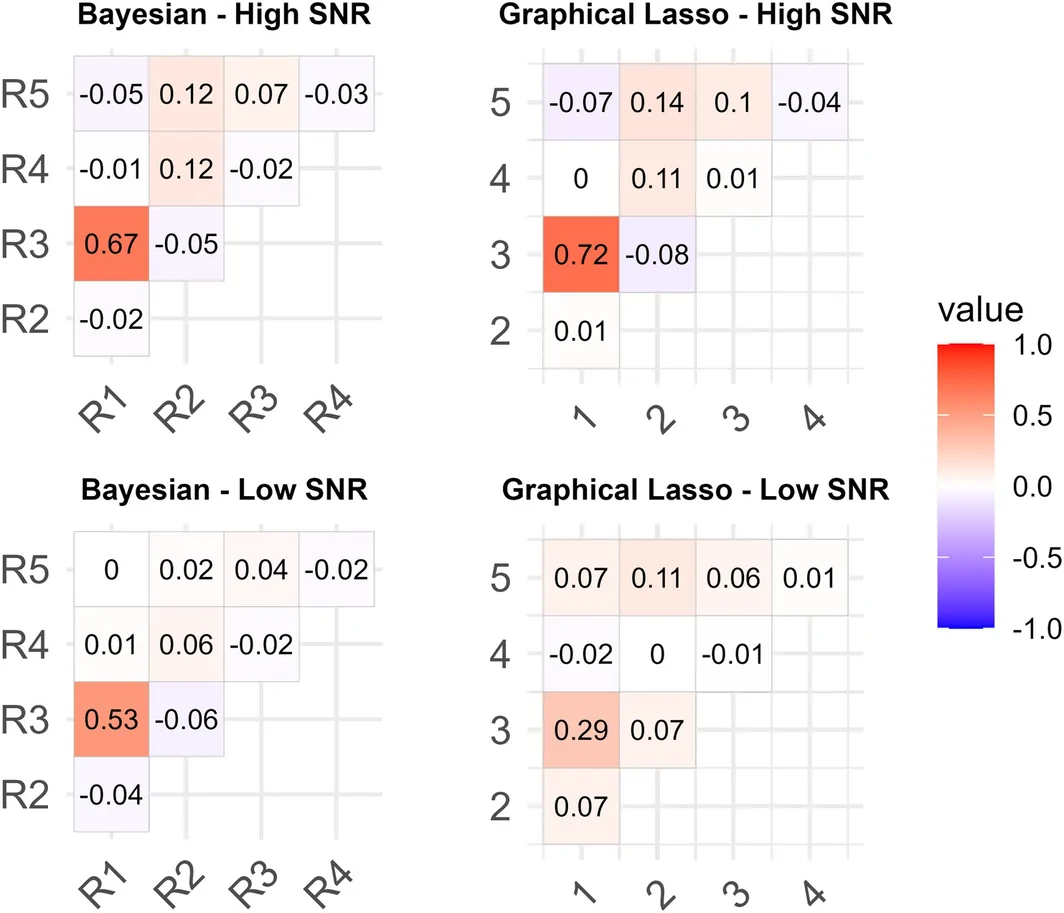
Estimates of the partial correlation for all possible region pairs using the Bayesian model and graphical lasso (regularization parameter = 0.1) under SNR = 10 (high) and SNR = 0.5 (low). The true partial correlation value for region pair (R1, R3) is set to 0.7 and the rest of the partial correlations are set to zero.

Our model also allows us to simultaneously infer connectivity between regions. We further compare the connectivity matrices estimated by our Bayesian model and the classical GLM in Figure 9. For the classical GLM approach, we compute functional connectivity in a seed‐wise manner. For each simulation seed, the regional time series are first rearranged such that regions serve as variables and subject‐time points are treated as observations. An empirical covariance matrix is then estimated across these observations, which is subsequently inverted to obtain the precision matrix. Pairwise partial correlations are derived from this precision matrix. These partial correlations quantify region‐to‐region dependencies conditional on all other regions. The final connectivity estimate is obtained by averaging the resulting partial correlation matrices across all simulation seeds to obtain a stable estimate. In addition to the OLS fitting approach for the classical GLM, we include a more modern two‐stage pipeline to provide a stronger baseline for comparison. In this approach, implemented using the glasso package in R, we first obtain residual time series and then apply graphical lasso to estimate a sparse precision matrix via an $\ell_{1}$‐regularized inverse covariance estimator, providing a fairer and more robust benchmark for connectivity estimation. The corresponding graphical lasso‐based connectivity matrices are presented in the Appendix in Figures B5 and B6 for two different regularization parameters (0.01 and 0.1). Notably, the results from graphical lasso are very similar to those of classical GLM in all SNRs conditions.

**FIGURE 10:**
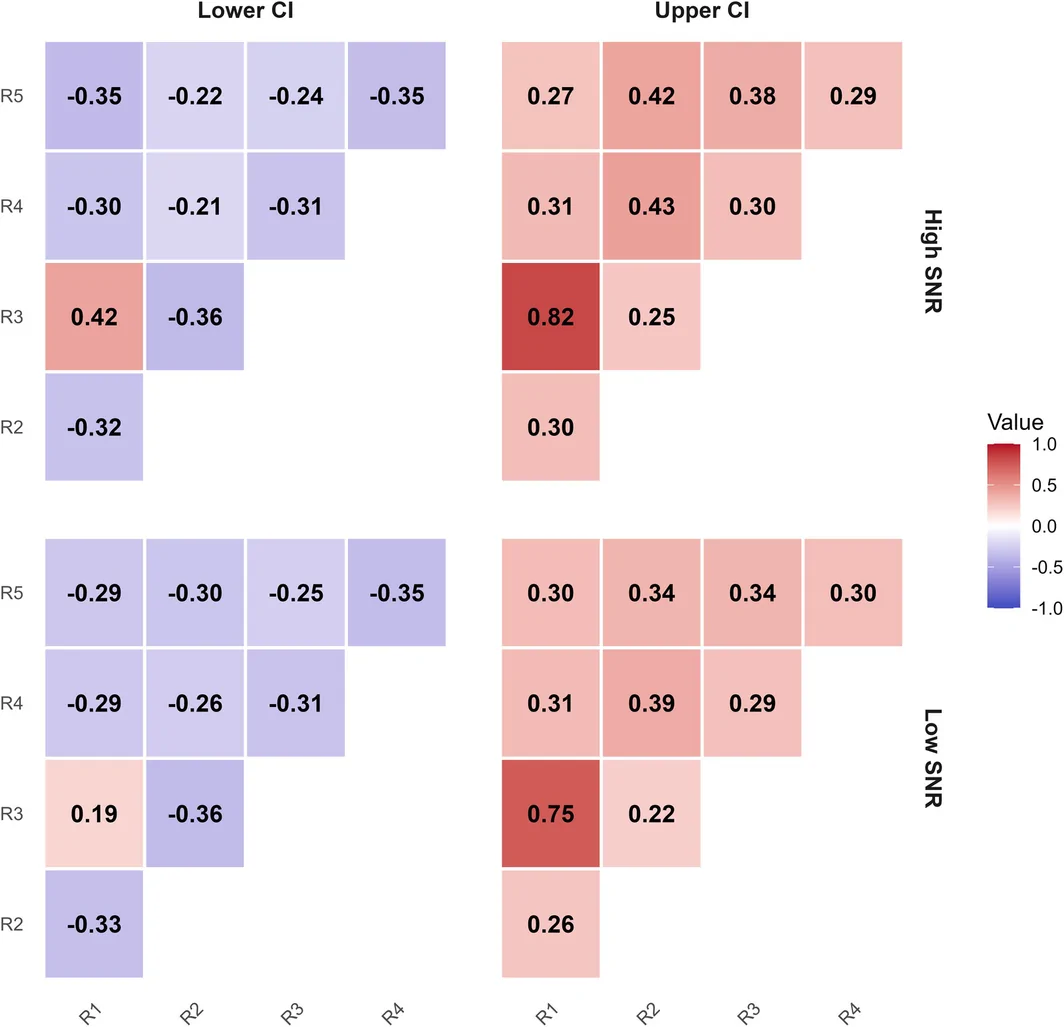
Comparison of uncertainty in partial correlation estimates obtained from the Bayesian model under high SNR (=10) and low SNR (=0.5). Each panel shows the posterior credible interval lower bound, and upper bound for all pairwise region combinations. Rows and columns correspond to regions R1–R5. The true partial correlation is nonzero only for region pair (R1,R3), with value 0.7, while all other true partial correlations are zero. Color scales are shared across all panels and SNRs.

In terms of the connectivity estimation for SNR=10, we see that classical GLM outperforms the Bayesian model in returning true correlation between regions 1 and 3 ($\rho_{13}$=0.7). However, the classical GLM overestimates the connectivity for disconnected regions, as there are also disconnected regions that are identified as connected. In that sense, the Bayesian model estimates smaller correlations compared to classical GLM. It is worth mentioning that in high‐SNR settings, the likelihood becomes highly informative, and the influence of the Graphical Lasso prior is naturally reduced through the data‐driven estimation of the shrinkage parameter ζ. As a result, no explicit adjustment of the prior is strictly necessary. Nevertheless, because the Laplace prior induces shrinkage toward zero, it may still introduce mild bias in estimating strong connections, as observed in Figure 9. In principle, one could reduce this bias by using a weaker shrinkage prior (e.g., smaller ζ or heavier‐tailed alternatives such as Student‐t priors). However, since the SNR is typically unknown in practice, adapting the prior based on SNR would introduce undesirable data dependence. We therefore retain the current hierarchical specification, which balances sparsity and robustness across different noise regimes. By moving to lower SNR, the Bayesian model demonstrates superior performance. The Bayesian model estimates that the region pair (R1,R3) has a partial correlation of 0.53 compared to an estimate of 0.32 from the classical GLM. Figure 10 shows the lower and upper bounds of the credible intervals for Bayesian partial correlation estimates under high‐ and low‐SNR conditions. The Bayesian credible intervals for most partial correlations include zero under both high‐ and low‐SNR conditions, indicating little evidence for robust nonzero associations, although the correlation between regions 1 and 3 remains consistently positive.

**FIGURE 11:**
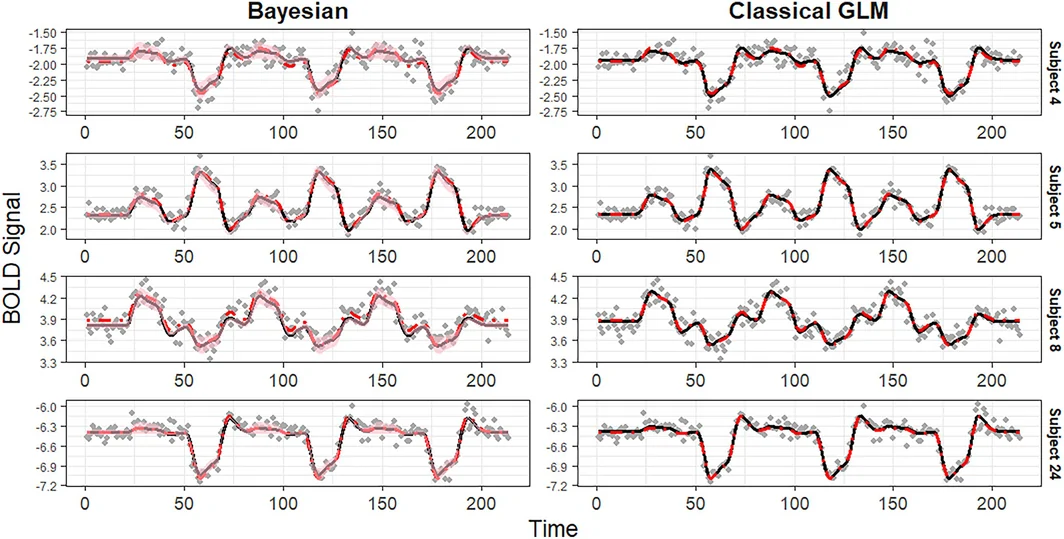
Model fit comparison for SNR = 10. The left column shows the model fit from the Bayesian model and the right column shows the model fit from the classical GLM. The dots in gray indicate the simulated response, the black solid line is the estimated response from the model, the red dash‐dot line indicates the estimated response for true values of parameters from the simulation study, and the pink shadow area represents the 95% credible interval.

**FIGURE 12:**
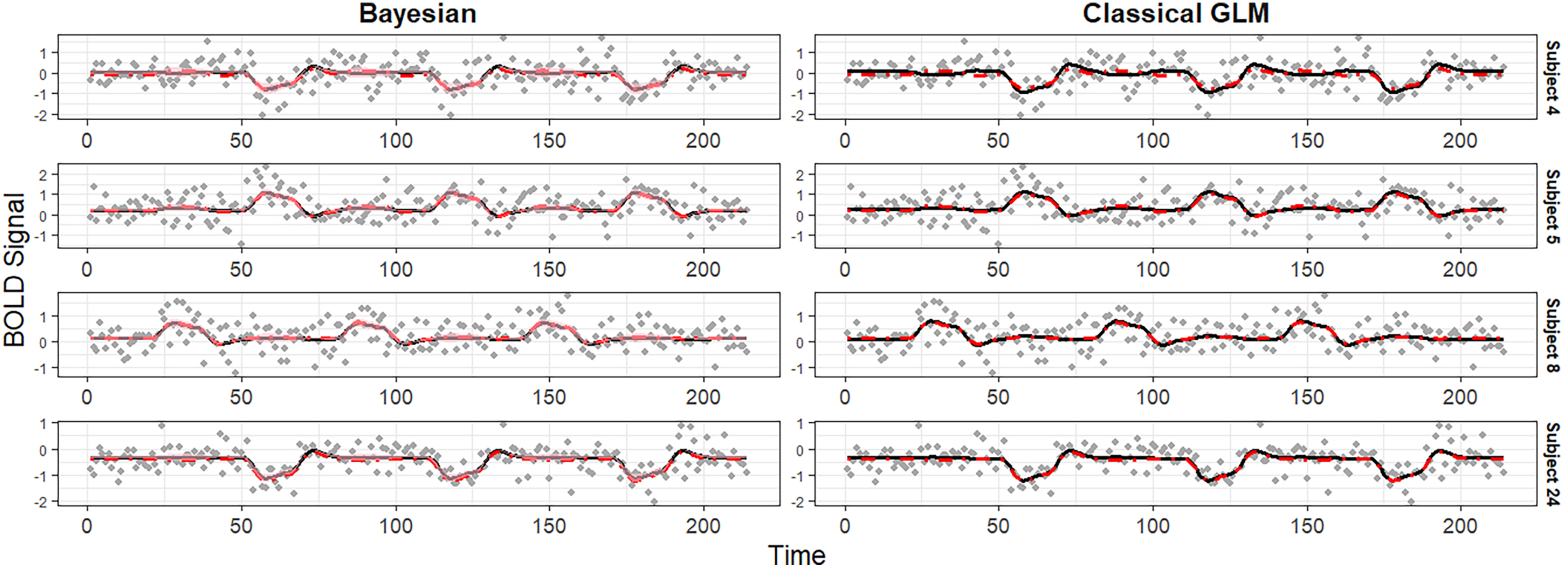
Model fit comparison for SNR = 0.5. The left column shows the model fit from the Bayesian model and the right column shows the model fit from the classical GLM. The dots in gray indicate the simulated response, the black solid line is the estimated response from the model, the red dash‐dot line indicates the estimated response for true values of parameters from the simulation study, and the pink shadow area represents the 95% credible interval.

**FIGURE B7:**
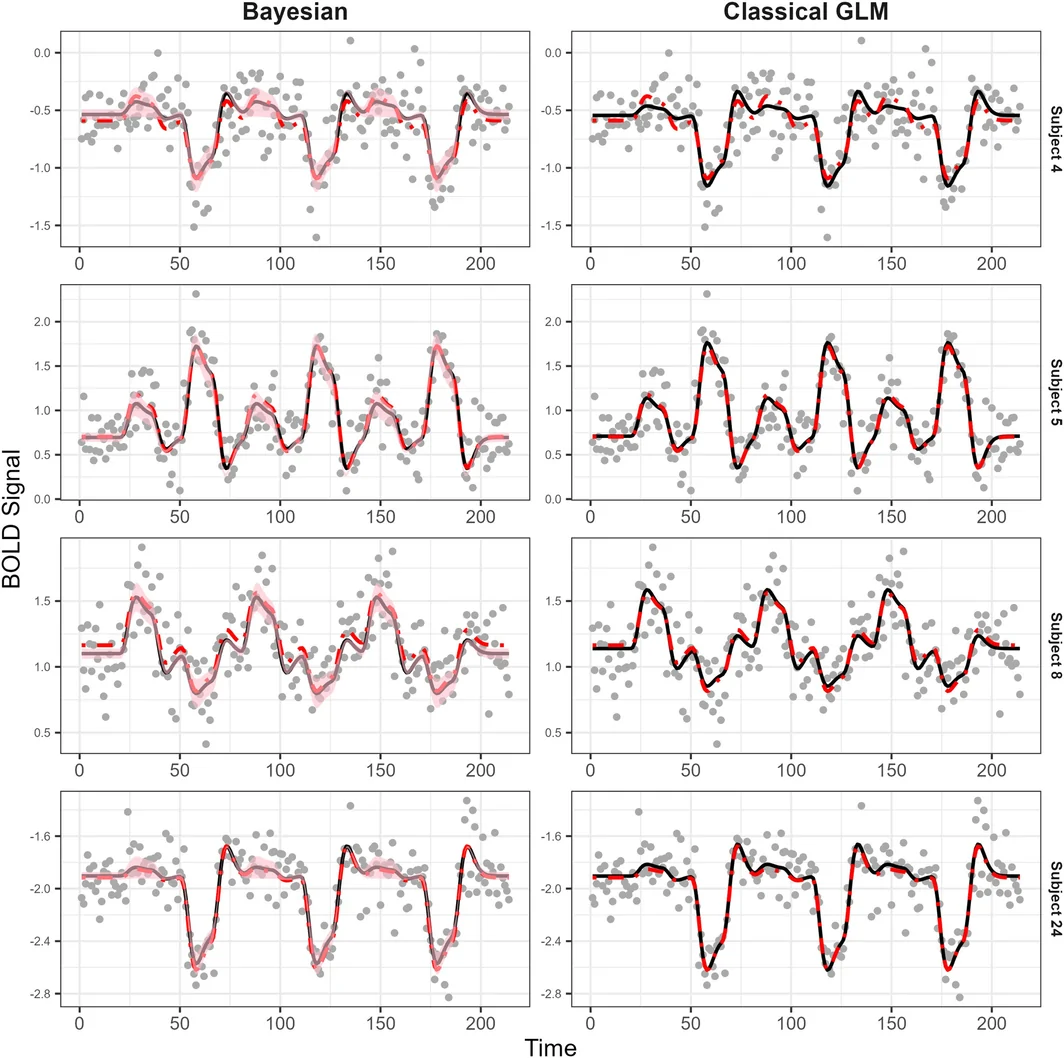
Model fit comparison for SNR = 3. The left column shows the model fit from Bayesian model and the right column shows the model fit from the classical GLM. The dots in gray indicate the simulated response, the black solid line is the estimated response from the model, the red dash‐dot line indicates estimated response for the true parameter values from the simulation study, and the pink shadow area represents the 95% credible interval.

**FIGURE B8:**
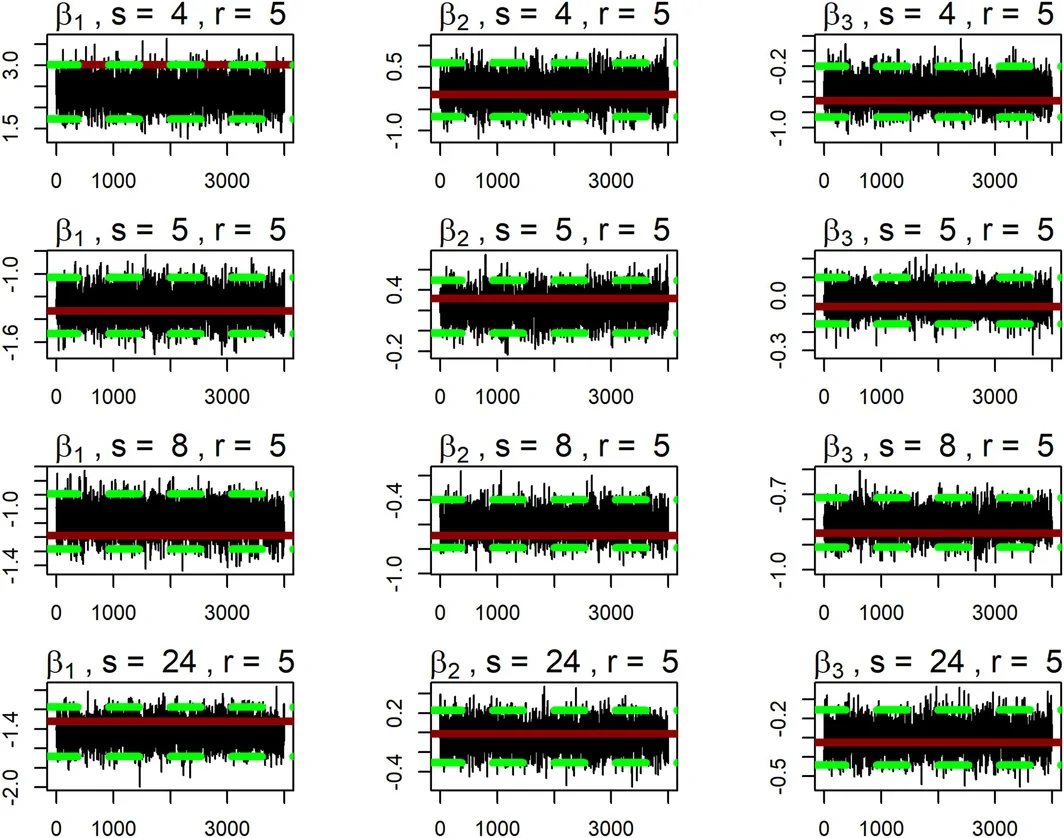
Trace plots for the β parameters of four different subjects and region 5 from MCMC sampling (SNR = 0.5). The chain runs for a total of 7000 iterations with the first 3000 as burn‐in period. Here we show only the iterations after the burn‐in period. The red line indicates the true parameter values from the simulation study, and the green‐dashed line presents the 95% credible interval.

**FIGURE B9:**
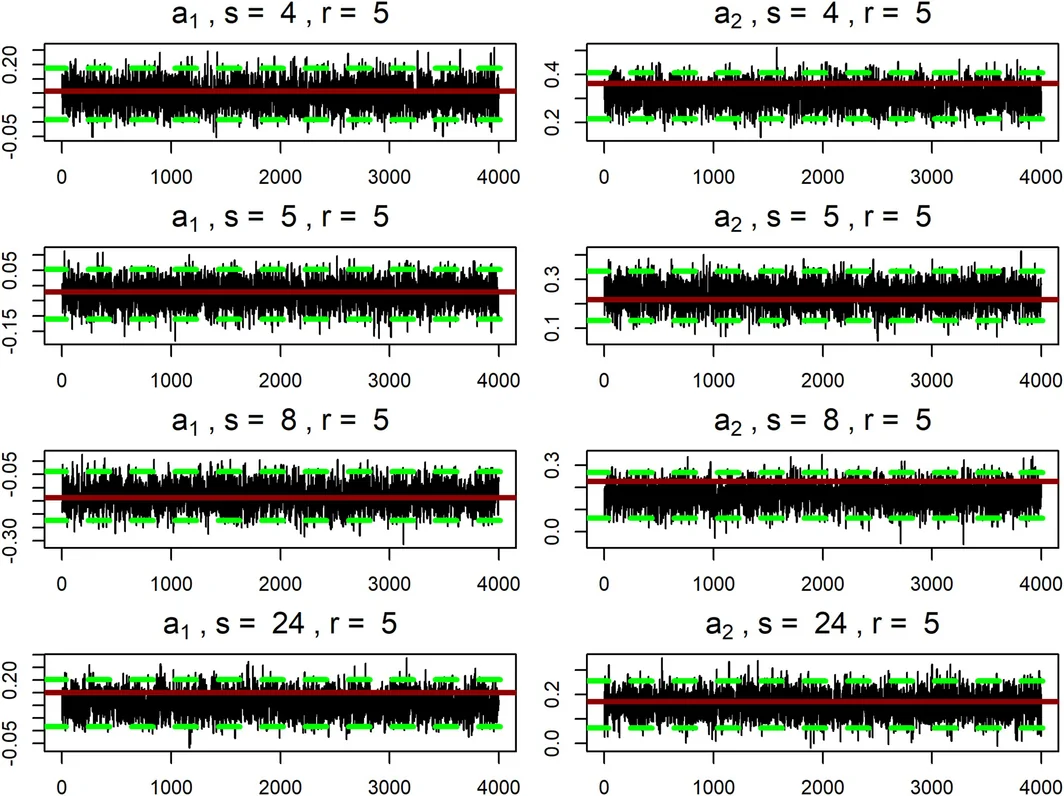
Trace plots for the a parameters of four different subjects and region 5 from MCMC sampling (SNR = 0.5). The chain runs for a total of 7000 iterations with the first 3000 as burn‐in period. Here we show only the iterations after the burn‐in period. The red line indicates the true parameter values from the simulation study, and the green‐dashed line presents the 95% credible interval.

**FIGURE B10:**
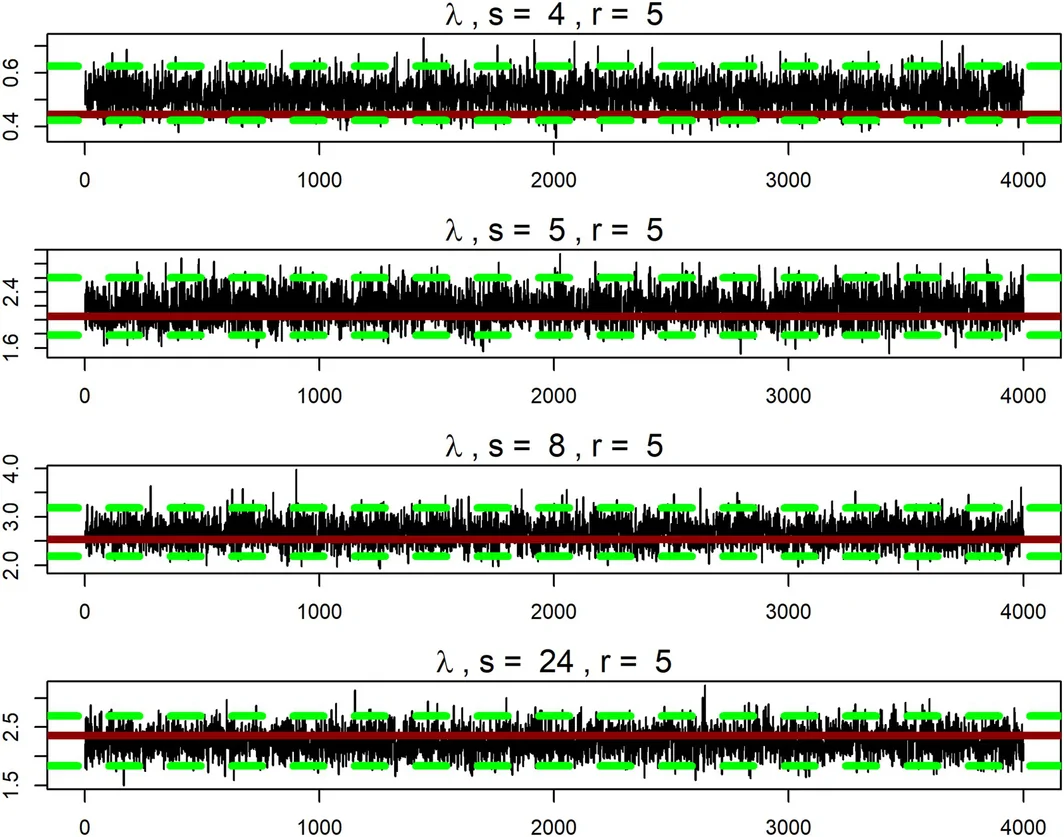
Trace plots for the $\lambda_{r}^{s}$ parameter of four different subjects and region 5 from MCMC sampling (SNR = 0.5). The chain runs for a total of 7000 iterations with the first 3000 as burn‐in period. Here we show only the iterations after the burn‐in period. The red line indicates the true parameter values from the simulation study, and the green‐dashed line presents the 95% credible interval.

**FIGURE B11:**
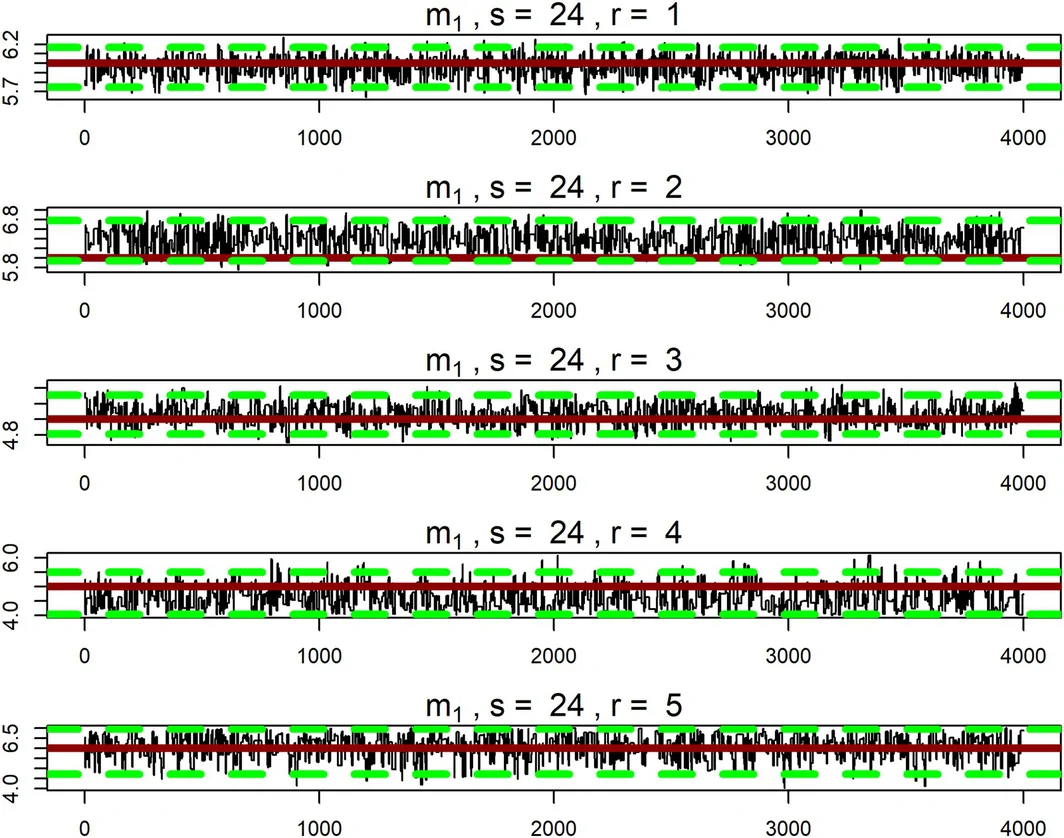
Trace plots for the $m_{1}$ parameter of subject 24 and five regions from MCMC sampling (SNR = 0.5). The chain runs for a total of 7000 iterations with the first 3000 as burn‐in period. Here we show only the iterations after the burn‐in period. The red line indicates the true parameter values from the simulation study, and the green‐dashed line presents the 95% credible interval.

**FIGURE B12:**
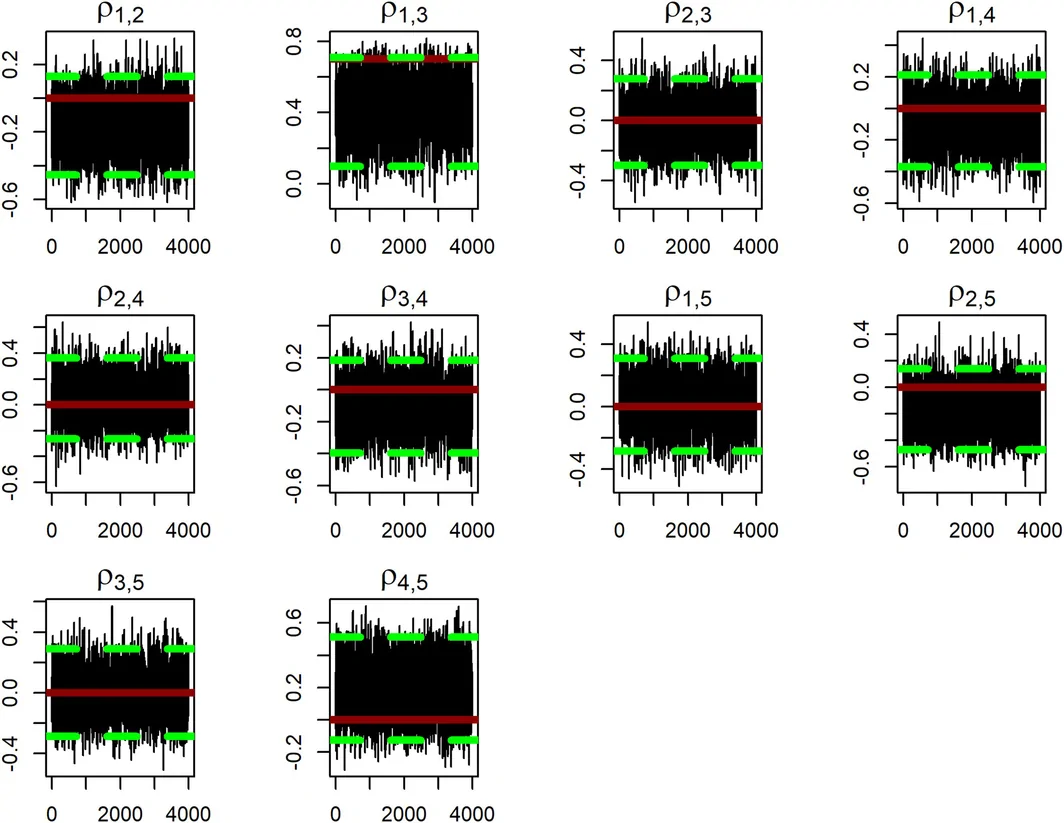
Trace plots for the partial correlation ρ between regions pair from MCMC sampling (SNR = 0.5). The chain runs for a total of 7000 iterations with the first 3000 as burn‐in period. Here we show only the iterations after the burn‐in period. The red line indicates the true parameter values from the simulation study, and the green‐dashed line presents the 95% credible interval.

Finally, we investigated the model fit obtained by the Bayesian model, and compared that to the one from classical GLM in low and high SNR scenarios. Figures 11 and 12 show the fit of the time series for four subjects. Once again, we notice a good fit for our Bayesian model, while the fitted curves are similar for both models. The model fit for moderate SNR(=3) is depicted in Figure B7 of Appendix B. Moreover, the trace plots of the posterior samples are shown in Figures B8, B9, B10, B11, B12 of Appendix B. The figures show the convergence of the MCMC chains for the different parameters in low SNR = 0.5 (the most difficult case).

## 5 Real Data Application

### 5.1 Human Connectome Project Dataset

The dataset used in this study was extracted from the Human Connectome Project (HCP) [44]. The 1200 Subjects Release (S1200) comprises behavioral and 3T MRI data from 1206 healthy young adults, collected between 2012 and 2015. The purpose of the HCP study is to identify key brain regions to support and interpret connectivity analyses from resting‐state fMRI, magnetoencephalography (MEG), and diffusion imaging. It also enables comparisons between task‐based and resting‐state connectivity and allows investigation of how activation patterns relate to individual differences in behavior and traits. To achieve this last goal, a diverse set of tasks was designed to involve a broad range of neural systems within realistic time constraints [45]. The HCP task battery was designed to assess seven major domains reflecting a broad spectrum of neural systems relevant to diverse research interests. These domains include:

1. visual, motion, somatosensory, and motor functions;
2. category‐specific representations;
3. working memory and cognitive control;
4. language processing, including semantic and phonological aspects;
5. social cognition, such as Theory of Mind;
6. relational processing; and
7. emotion processing.

In this study, we focus on the motor task fMRI data from a subset of 25 subjects. However, the proposed approach can be applied to other tasks as well. The acquisition parameters for the HCP task fMRI data are as follows: repetition time (TR) = 720 ms, echo time (TE) = 33.1 ms, flip angle = 52°, bandwidth (BW) = 2290 Hz/Px, in‐plane field of view (FOV) = 208 × 180 mm, with 72 slices and a spatial resolution of 2.0 mm isotropic voxels. During the motor task, participants received visual cues instructing them to tap either their left or right fingers, squeeze their left or right toes, or move their tongues. Each functional run includes 13 blocks: two blocks each for left hand (LH) and right hand (RH) movements, two for left foot (LF) and right foot (RF) movements, two for tongue (T) movement, and three fixation blocks, each lasting 15 s. Every task block spans 12 s and is preceded by a 3‐second visual cue. Each run comprises 284 time frames and has a total duration of 214 s. Preprocessing of the data follows the HCP minimal preprocessing pipeline, which includes steps such as motion correction, slice timing correction, spatial smoothing, high‐pass temporal filtering, and nonlinear registration using FNIRT to the MNI152 standard space. More details regarding the data acquisition and experimental design are available in Barch et al. [45].

**FIGURE 13:**
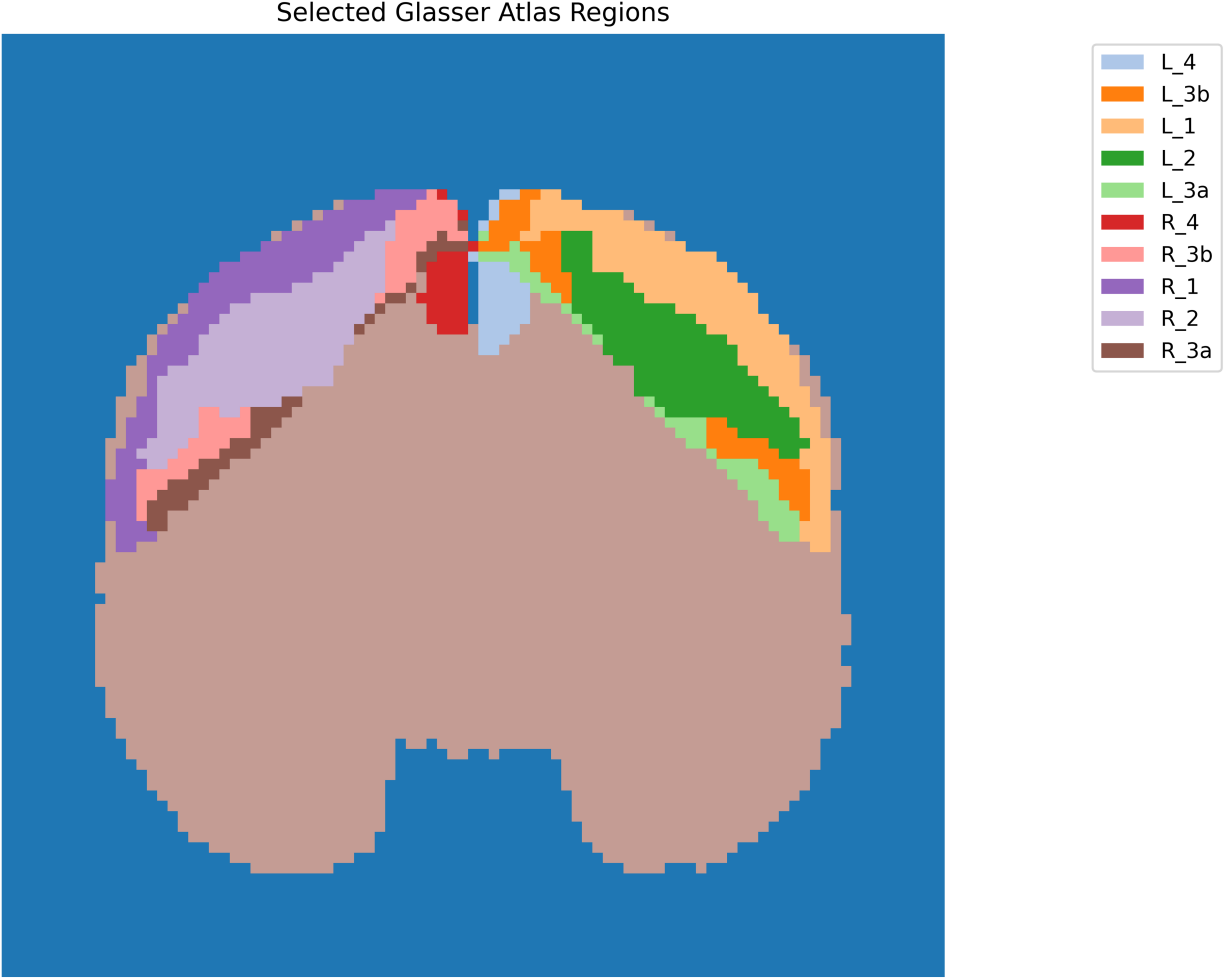
Ten selected ROIs from the motor and somatosensory cortex. The prefixes L and R denote the left and right hemispheres, respectively. Area 4 corresponds to the primary motor cortex, while areas 1, 2, 3a, and 3b represent subdivisions of the primary and secondary somatosensory cortices.

For the data preparation stage, we constructed subject‐specific design matrices by including all five stimuli from the motor task. For each subject and each brain region, a separate design matrix was generated using the canonical double gamma HRF parameterized with region‐specific peak delay values (5 s and 6 s). The event onset times and durations were filtered to remain within the total duration of the scan with the HRF at the subject‐specific TR. This produced a 3D tensor per subject (region × time × task condition), used to model the expected BOLD response under experimental conditions. We initialized the HRF parameter in the MCMC algorithm with $m_{1}=5.5s$ (the same value for each region). The HCP‐MMP1.0 atlas [46] was resliced using SPM [37] to match the voxel resolution and dimensions of the preprocessed functional images in MNI space. In the HCP‐MMP1.0 atlas, labels L_4, L_3b, L_1, L_2, L_3a, (and their right hemisphere counterparts) refer to cortical areas within the sensorimotor system. Specifically, area 4 corresponds to the primary motor cortex, while areas 1, 2, 3a, and 3b represent subdivisions of the primary and secondary somatosensory cortices. The prefixes L_ and R_ denote the hemispheres (left and right, respectively). These regions were identified based on a combination of anatomical, functional, and connectivity features, providing a high‐resolution parcellation of the human cortex. The selected regions correspond to the HCP motor task paradigm [45], which are shown in Figure 13.

We extracted regional BOLD signals using a parcellation‐based approach [46]. For each region, we computed the mean time series by averaging the BOLD signal across all voxels assigned to that parcel, as defined by the atlas. To reduce the influence of low‐frequency drifts and physiological noise, we performed temporal denoising by projecting out a set of nuisance regressors consisting of polynomial (linear, quadratic, and cubic) and sinusoidal (sine and cosine) trends at one and two cycles per scan duration. These nuisance components were regressed out from the time series using Ordinary Least Squares (OLS). The result is a temporally filtered time series. The final cleaned and preprocessed output is a three‐dimensional response tensor $Y\in {\mathbb{R}}^{S\times T\times R}$, where S is the number of subjects, T is the number of time points (with T=284), and R=10 corresponds to the selected ROIs.

The previous study by Barch et al. [45] concludes that limb control is primarily contralateral, while midline effectors such as the tongue involve more symmetric cortical engagement. They hypothesize that motor‐related β responses in the primary somatosensory and motor regions reflect a contralateral dominance for unilateral limb movements and bilateral engagement for midline actions. In this study, we analyze the data to verify these conclusions and assess functional connectivity between the defined regions of interest. Our aim is to show that our model will recover activations in cortical areas within the sensorimotor system, and that there will be some positive partial correlations between ROIs. To achieve this aim, the proposed Bayesian model was fitted to the data extracted from ROIs in the motor and somatosensory cortex.

**FIGURE 14:**
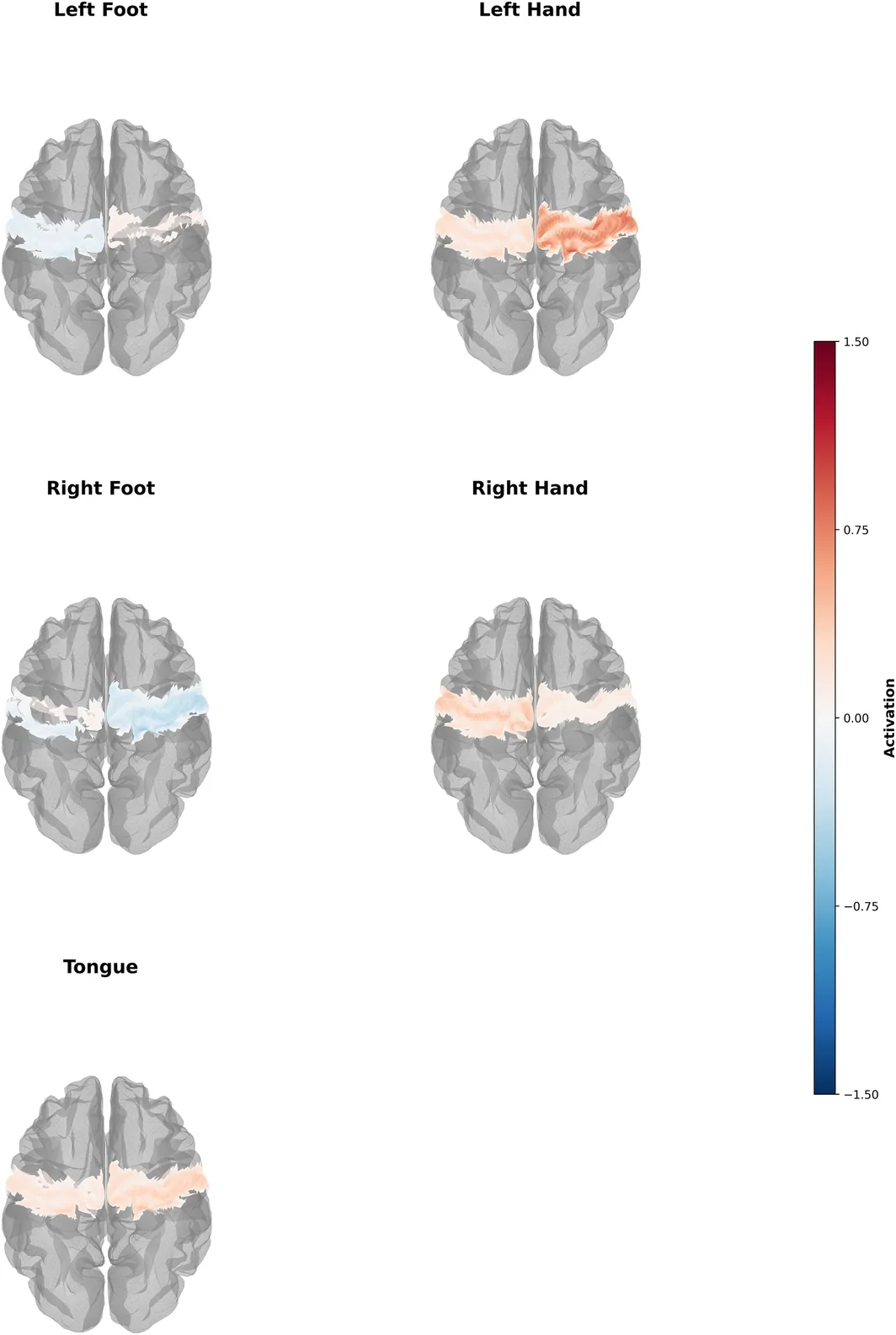
Estimated β activations from the Bayesian model for five stimuli from subject 21 in ten ROIs.

Due to the large size of the data, 10,000 samples were drawn from the joint posterior distribution of all parameters, and the first 2000 samples were discarded as ‘burn‐in’. In addition, the effectiveSize function within the coda package in R is used to calculate the median values for the effective sample size for the 10,000 posterior draws of the parameters. The median effective sample sizes were as follows: 4024 for β; 3121 for a; 804 for $m_{1}$; 4794 for λ; 1002 for Ω. These values suggest a good convergence of the chain. It is worth mentioning that the relatively slower mixing observed for Ω is consistent with general MCMC theory, which suggests that parameters subject to strong posterior dependence and hierarchical structure often exhibit greater autocorrelation and lower effective sample sizes than simpler scalar parameters [47]. In Bayesian Graphical Lasso models, these challenges may be compounded by the positive‐definiteness constraint on Ω. The median of the posterior samples of β was calculated as the final estimate of brain activation. The activation maps of subject 21 are shown in Figure 14. Higher values of the coefficient suggest that there is an increase in the BOLD response associated with the task.

The β activation patterns observed for five motor tasks (left foot, left hand, right foot, right hand, and tongue) demonstrate expected lateralization consistent with the contralateral organization of the motor system. Specifically, movements of the left limbs (foot and hand) show predominant activation in the right hemisphere regions R_4, R_3b, while right limb movements elicit activation in the corresponding left hemisphere regions L_4, L_3b, L_1. In contrast, movement of the tongue, a midline function, results in more bilateral activation, with moderate responses in both hemispheres. These patterns align with established neurophysiological findings that limb control is primarily contralateral, while midline effectors such as the tongue involve more symmetric cortical engagement [45]. These results support the expected functional lateralization of the motor system in individual‐level β activation profiles.

**FIGURE 15:**
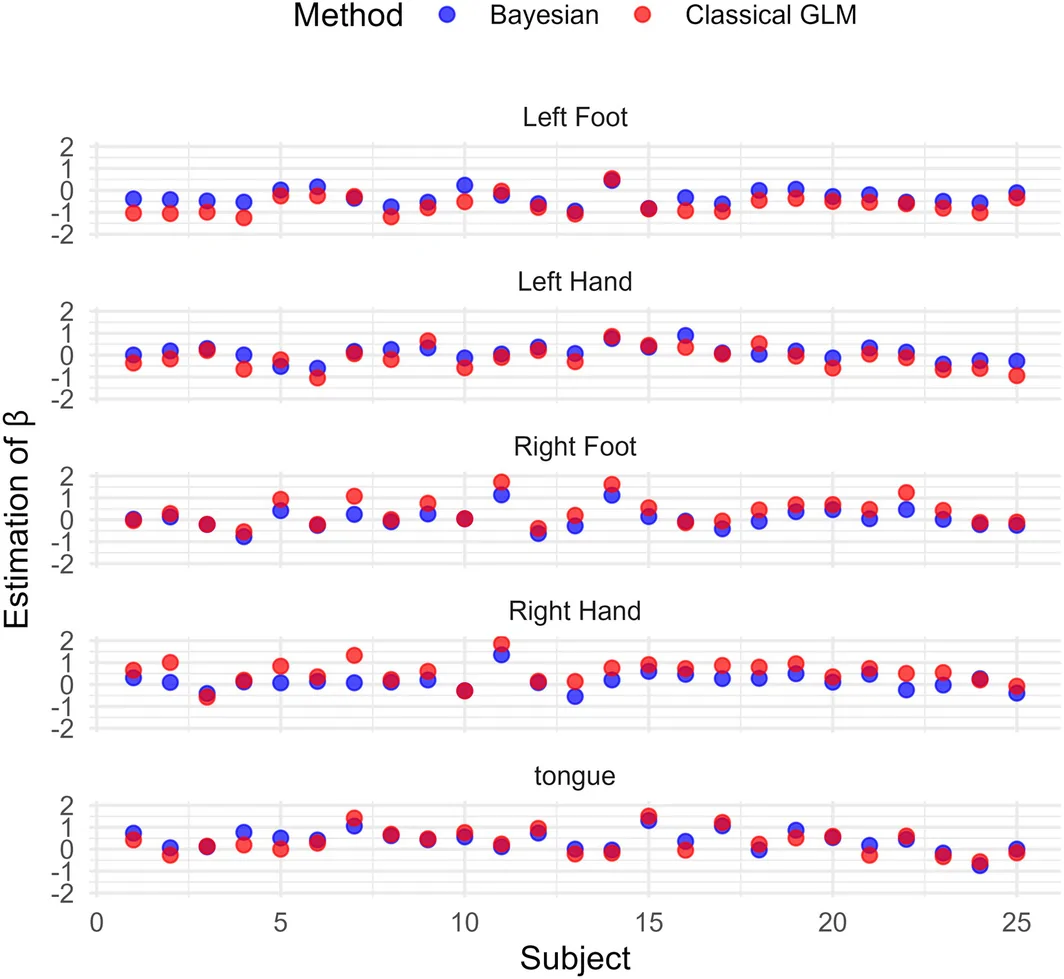
Estimated β activations for the first region (all subjects, all five stimuli). The blue dots correspond to the Bayesian model, while the red dots correspond to the classical GLM.

To show the performance of the model, the estimation of β from both the Bayesian model and the classical GLM is presented in Figure 15 for the first region (all subjects, all five stimuli). The results obtained from the two approaches are similar.

**FIGURE 16:**
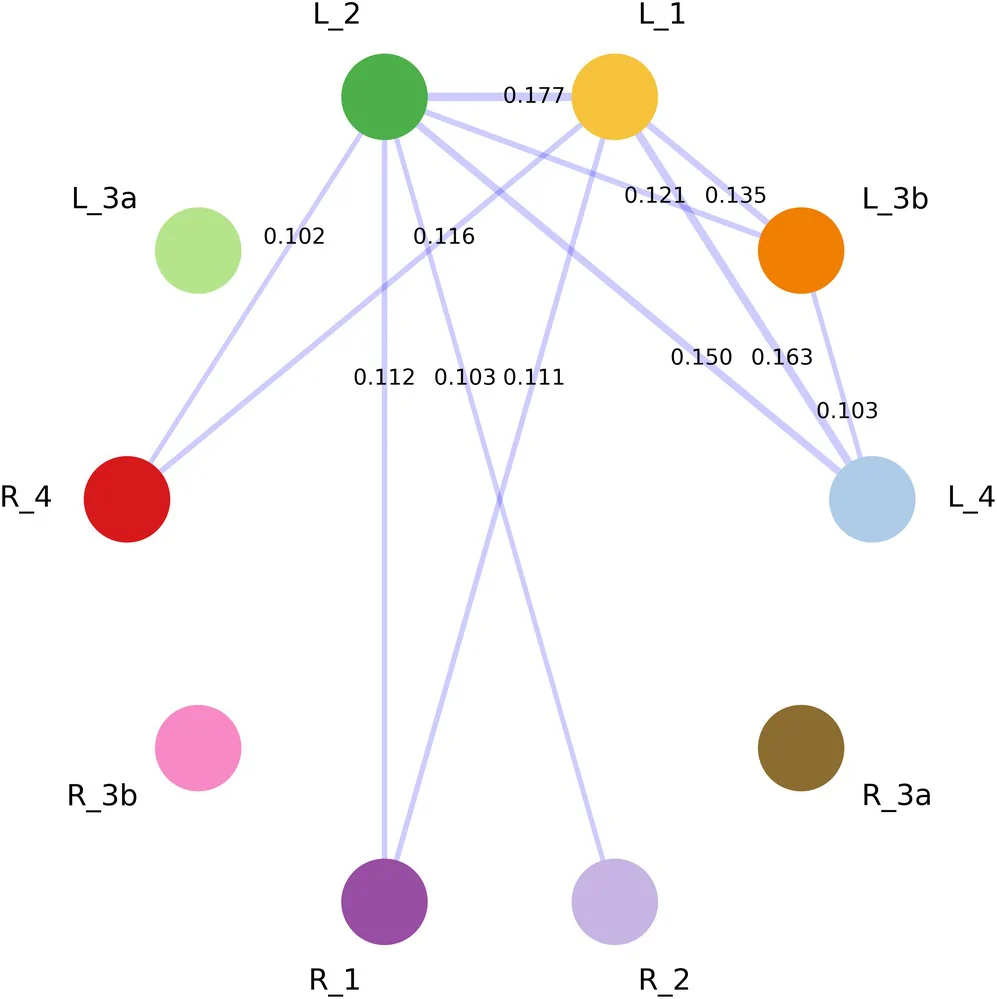
Graph showing the connected regions of the brain, based on the partial correlation from the Bayesian model. Here, we set the threshold for connectivity at 0.1 correlation.

Estimates for significantly nonzero partial correlations between regions are shown in Figure 16. The partial correlation values are low, which is expected due to the high noise level and smoothing in the data, and also due to the strong regularization imposed by the Gaussian graphical prior. The strongest edges were observed between L_1 and L_2 (ρ=0.177) and L_1 and L_4 (ρ=0.163), indicating strong intrahemispheric functional coupling within the left hemisphere. These regions are part of the primary motor cortex (M1) and primary somatosensory cortex (S1), which are known to co‐activate during unilateral movements, especially those involving the right limbs [48, 49]. Lower interhemispheric connections were observed, such as between L_2 and R_4 (ρ=0.102) and L_2 and R_1 (ρ=0.112), suggesting bilateral coordination during motor activity [50]. Regions such as R_3a, R_3b, and L_3a appeared relatively disconnected in the network, which may reflect their more specialized roles in proprioception or passive somatosensory input not primarily engaged in the executed motor task. This is consistent with the known functional segregation within the somatosensory system [51].

**FIGURE 17:**
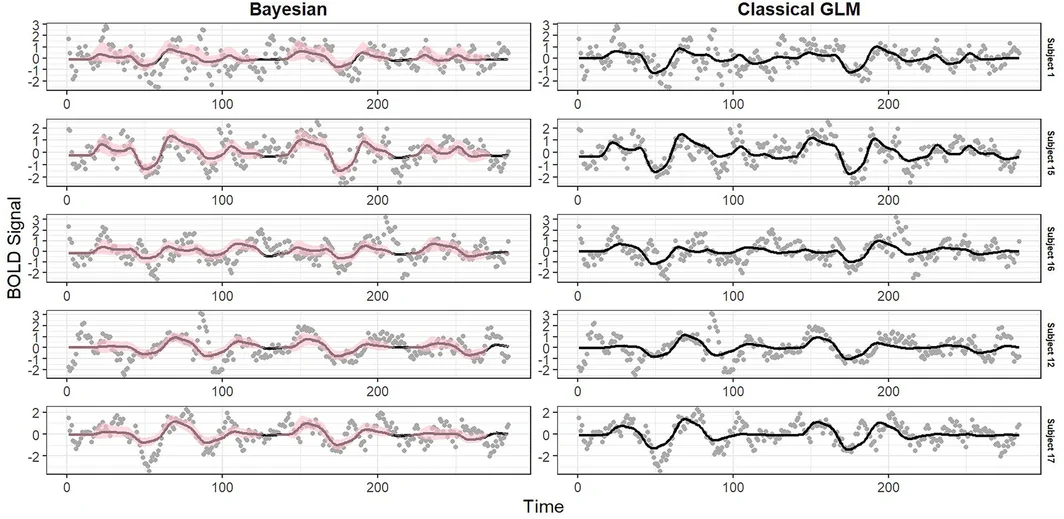
Model fit comparison for task fMRI data. The left column shows the model fit from the Bayesian model and the right column shows the model fit from the classical GLM. The dots in gray indicate the response, the black solid line is the estimated response from the model, and the pink shadow area represents the estimated 95% credible interval.

In general, the observed network structure supports previously reported patterns of lateralized activation and connectivity during motor tasks [52, 53]. The strong intrahemispheric connectivity in the left hemisphere is consistent with contralateral (right‐limb) motor control, while the interhemispheric links support coordination between hemispheres as required by the task. Finally, we show the model fit from both Bayesian and GLM in Figure 17, which indicates a similar fit for both approaches. However, we can also quantify the uncertainty from the Bayesian model, which is not the case for classical GLM.

### 5.2 Individual Brain Charting Dataset

To evaluate the robustness and generalizability of the proposed Bayesian model, we also tested it on an independent dataset. The second dataset used in this study was obtained from the preprocessed 3‐mm version of the Individual Brain Charting (IBC) dataset [54], available through EBRAINS. The IBC project is a high‐resolution 3T functional MRI dataset designed for cognitive mapping and includes data from 13 healthy participants who completed a large battery of cognitive tasks under highly standardized acquisition conditions. The repeated measurement of the same individuals across all experimental conditions reduces inter‐subject variability and enables fine‐grained investigation of functional brain organization within individuals.

For the present study, we selected the *ArchiStandard* protocol, which probes a broad range of cognitive functions, spanning sensory, motor, language, and higher‐order cognitive processes. The protocol employs a fast event‐related design with ten experimental conditions:

1. visually cued left‐hand button presses;
2. visually cued right‐hand button presses;
3. auditorily cued left‐hand button presses;
4. auditorily cued right‐hand button presses;
5. auditory sentence comprehension;
6. sentence reading;
7. viewing flashing horizontal checkerboards;
8. viewing flashing vertical checkerboards;
9. silent subtraction following visual instructions; and
10. silent subtraction following auditory instructions.

Data from all 13 participants acquired during the first experimental session were included in the analysis. We focused on runs corresponding to fast event‐related paradigms involving well‐defined cognitive operations. Additional details regarding the experimental design and data acquisition procedures are provided by [54].

Functional images were preprocessed using the standard IBC pipeline and resampled to 3‐mm isotropic resolution in MNI space. Regional time series were extracted using the HCP‐MMP1.0 cortical parcellation atlas [46]. To ensure spatial correspondence with the functional data, the atlas was resliced in SPM12 [37] to match the image dimensions and voxel resolution.

Based on prior neuroanatomical knowledge, we selected ten ROIs spanning motor, auditory/language, visual, and executive networks: L_4, L_6d, L_6ma, L_A1, L_PSL, L_44, L_45, L_V1, L_V2, and L_IPS1. Mean BOLD time series were obtained by averaging voxel intensities within each parcel. To reduce low‐frequency drift and physiological noise, we regressed out polynomial (linear, quadratic, and cubic) and sinusoidal (sine and cosine) trends at one and two cycles per scan using OLS. This produced temporally filtered time series. The final preprocessed data are represented by a response tensor $Y\in {\mathbb{R}}^{S\times T\times R}$, where S=13 is the number of subjects, T=156 is the number of time points, and R=10 is the number of selected ROIs. The model was subsequently used to estimate both task‐evoked activation and functional connectivity.

**FIGURE D1:**
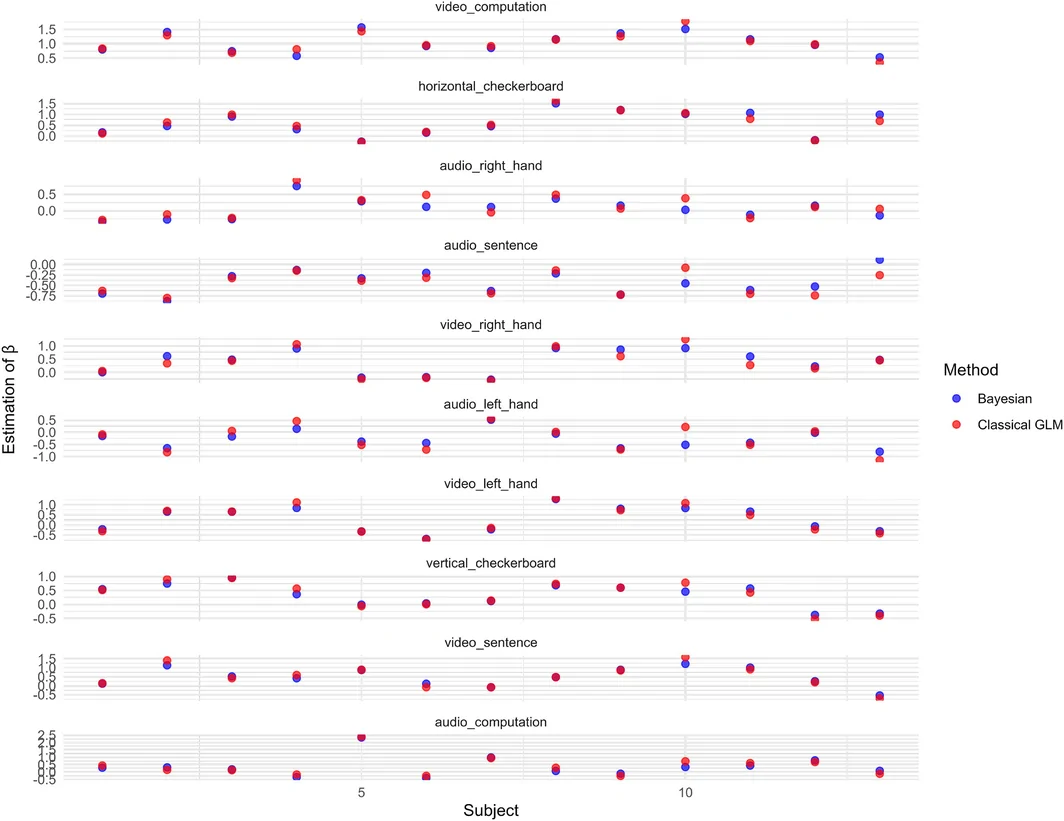
Estimated β activations for the first region (all subjects, all five stimuli). The blue dots correspond to the Bayesian model, while the red dots correspond to the classical GLM.

**FIGURE D2:**
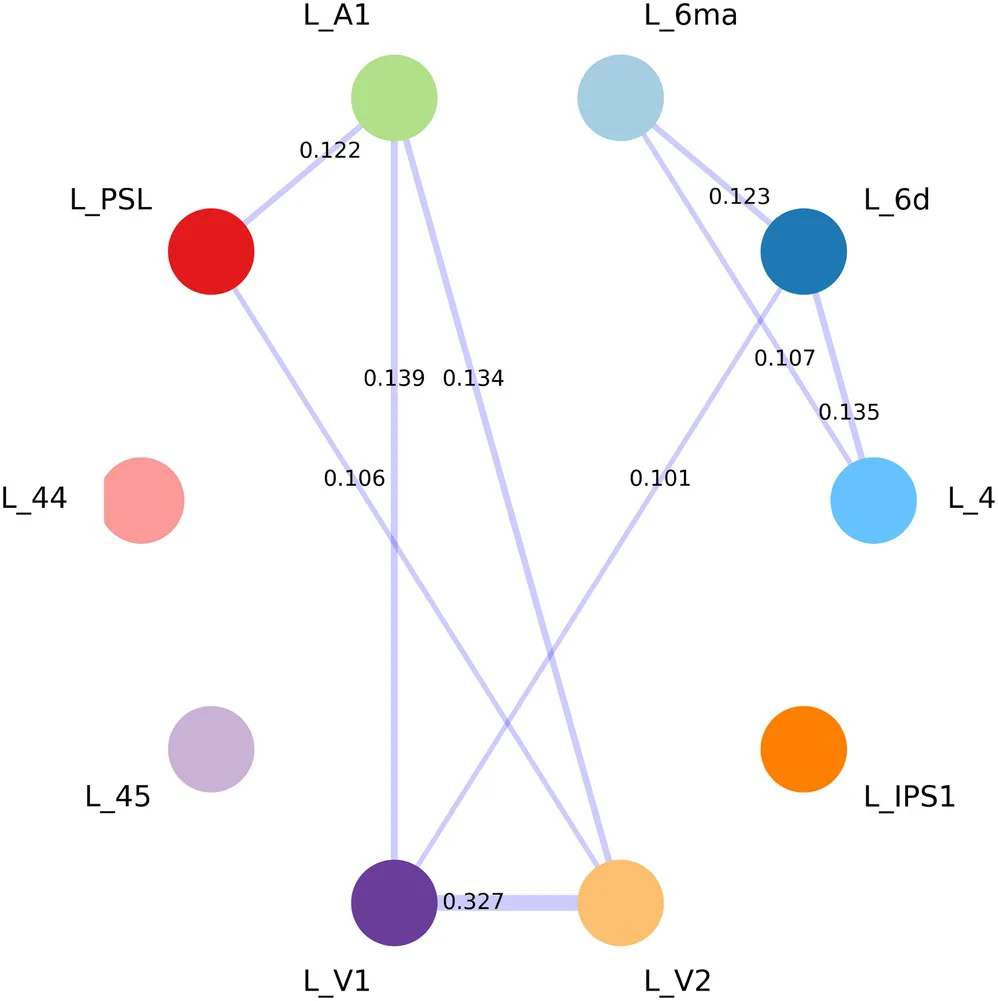
Graph showing the connected regions of the brain, based on the partial correlation from the Bayesian model. Here, we set the threshold for connectivity at 0.1 correlation.

**FIGURE D3:**
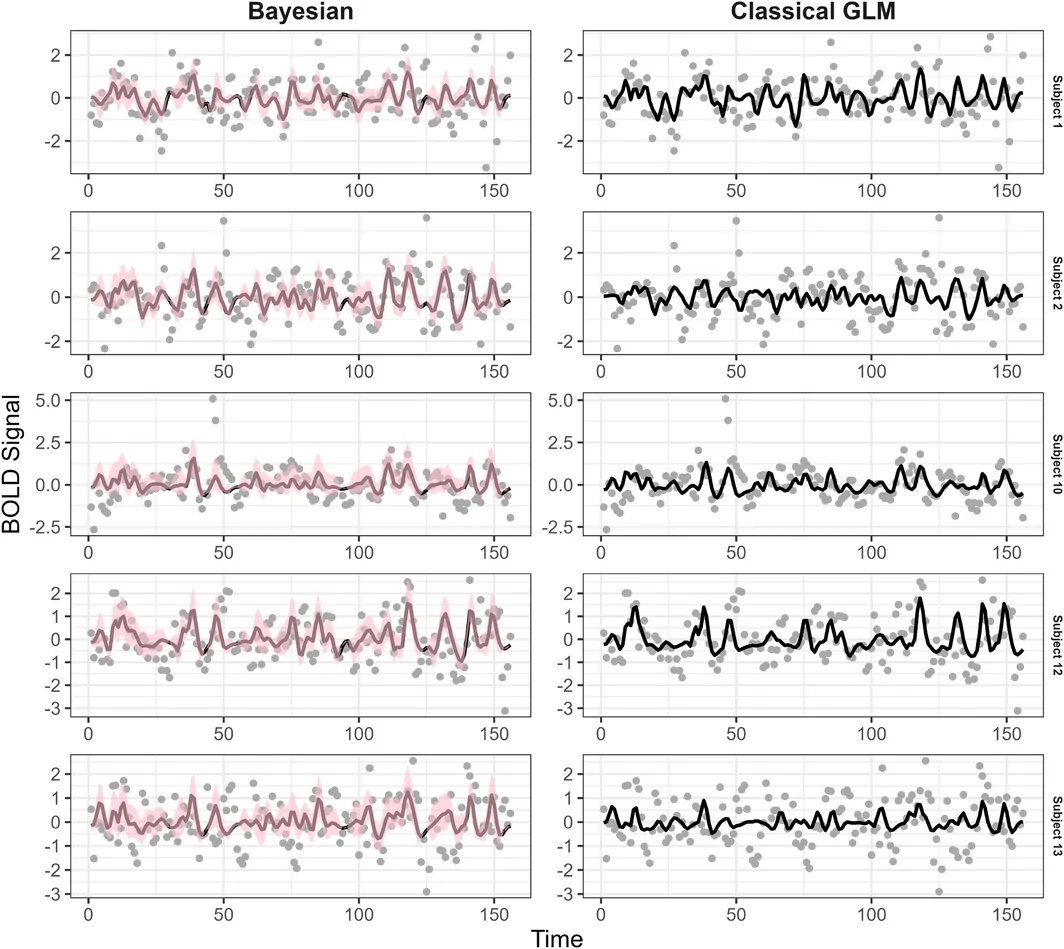
Model fit comparison for task fMRI data. The left column shows the model fit from the Bayesian model and the right column shows the model fit from the classical GLM. The dots in gray indicate the response, the black solid line is the estimated response from the model, and the pink shadow area represents the estimated 95% credible interval.

For validation, activation estimates obtained from the Bayesian model were compared with those derived from classical GLM. In addition, overall model fit was evaluated for both approaches. The two methods yielded highly consistent activation patterns, supporting the validity of the proposed framework. Detailed results and comparative analyses are provided in Figures D1, D2, D3 of Appendix D.

## 6 Discussion and Future Work

This paper presents a unified and probabilistically consistent framework for analyzing task‐related brain activity across multiple subjects in fMRI studies. Our model has several advantages:

1. it is able to simultaneously infer activation and connectivity;
2. it estimates HRFs for each region;
3. it demonstrates increased robustness to noise compared to the standard classical GLM.

The proposed model builds on the vast literature on spatiotemporal linear regression models, extending the GLM‐AR framework introduced in several works [30, 31, 32, 41, 55]. In this formulation, the UGL prior is applied over both the regression and autoregressive coefficients to capture spatial structure. The idea of functional connectivity is inspired by Spencer et al. [29], who incorporated a random effect term and used a Bayesian Graphical Lasso prior to infer connectivity between brain regions. In the present study, we build on these methodological advances to formulate a unified framework that allows simultaneous estimation of activation and connectivity.

Our strategy focuses on examining brain activation in predetermined ROIs. To manage the high‐dimensionality of fMRI data, we partition the whole brain into ROIs that can be defined in terms of structural or functional features. This approach, commonly used in fMRI analyses [46], enables more efficient estimation while preserving meaningful regional information for both activation and connectivity inference. To show the performance of the proposed model, we compared it to the standard classical GLM. Simulation studies showed our model outperforms classical GLM in terms of both activation and connectivity, especially in the scenario with low SNR. Although there is no ground truth in the real data, we compared the results of our model to previous studies on HCP motor task data sets. The results showed activations in cortical areas within the sensorimotor system, and some partial correlations between ROIs, which is consistent with previous studies [45, 52, 53].

A potential future improvement for this research is to extend our Bayesian model to explicitly account for between‐subject heterogeneity in neuronal activity by incorporating a spatially informed, multisubject nonparametric variable selection prior [56]. Such an extension could further enhance the model's power by effectively borrowing information across subjects. Another limitation of the current model is that we did not consider the between‐session effects [57]. For real data analysis, we only used one session for simplicity, as the activation strength may vary across the sessions. In future studies we will extend our model to consider the variation in the activation strength among different sessions.

Our current model estimates activation at the ROI level, but in the future, we could extend the model to infer activation at the voxels level. One way to implement this would be to use tensor regression. Frequentist and Bayesian approaches for joint modeling of the BOLD response across all voxels in the form of a tensor have also been proposed [58, 59]. In the current study, we employed MCMC sampling algorithms. While MCMC is generally more accurate than approximate methods, it may not be sufficiently efficient for practical applications involving whole‐brain datasets with a large number of conditions. To improve scalability, alternative approaches such as Variational Bayes (VB) and Integrated Nested Laplace Approximation (INLA) could be considered [60, 61]. For example, variational inference approaches have been successfully developed within the JDE framework to improve scalability in high‐dimensional fMRI settings [62]. Another approach is to use the Preconditioned Conjugate Gradient (PCG) based Gaussian Markov Random Field (GMRF) sampling, which offers an efficient method for performing exact inference in high‐dimensional spatial models, such as those used in task‐based fMRI studies [55].

When it comes to HRF modeling, the nonlinearity and constraints inherent in the double‐gamma parameterization, together with well‐known identifiability challenges, required us to fix four HRF parameters and estimate only one, namely the delay of the response ($m_{1}$). This parameter has been shown to vary substantially across fMRI studies [4]. While this simplification improved the stability of posterior inference, it is also a limitation of the current work, as it restricts the range of HRF shapes that can be represented. Attempts to jointly estimate all double‐gamma parameters using MCMC methods often caused the Metropolis–Hastings sampler to become trapped in local optima.

A variety of alternative HRF representations have been proposed, including nonlinear parameterizations such as gamma, cosine, and inverse‐logit functions [6], as well as linear approaches such as spline bases, finite impulse response (FIR) models [19, 62, 63], and semi‐parametric methods [64]. In particular, FIR‐style HRF estimation with temporal smoothness priors has been developed within the JDE framework [18], avoiding many of the identifiability issues associated with the double‐gamma parameterization. A natural middle ground is provided by constrained linear basis functions, [65], which offer greater flexibility than fixed parametric HRFs while remaining computationally efficient. Incorporating such approaches into the proposed framework represents an important direction for future work.

Other work has extended Bayesian deconvolution methods to resting‐state fMRI. For example [66], proposed a multivariate semi‐blind deconvolution framework that jointly estimates latent neural activity and HRFs without requiring task onsets, providing an alternative strategy for addressing HRF identifiability in high‐dimensional settings. Moreover, we note that an important limitation of the current model is that all but one of the HRF parameters are fixed, while we assume that the estimated parameter $m_{1}$ has the same value for each subject. As a result, the model is not able to perfectly capture greater variability in individual‐subject HRF, even under relatively high SNR. Incorporating hierarchical priors on the HRF parameters represents a natural direction for future work and could improve estimation precision while preserving subject‐specific differences [4, 21].

In the current study, we only considered functional connectivity; a future research line would be to infer causal connectivity. Causal connectivity, often referred to as Effective Connectivity ‐, describes the directed interactions among distinct brain regions [67] and is used to understand how activity in one brain region influences activity in another, revealing the flow of information across the brain. However, the performance of current methods can be limited by practical challenges. One major issue is the low temporal resolution of fMRI, which can lead to uncertainty in the results. This may cause problems such as low reproducibility in identified connectomes and reduced precision [68, 69]. As shown by Eggeling et al. [70], incorporating prior knowledge that takes into account Diffusion Tensor Imaging (DTI) signals [71] can significantly improve the accuracy and reproducibility of inferred graphs, especially when data are limited (e.g., fMRI signals) and the network dimensionality increases. Therefore, developing Bayesian causal approaches that use multimodal data, such as incorporating DTI as a prior, may offer a way to address the challenges mentioned above. Moreover, deconvolution‐based approaches estimate latent neural signals from BOLD data without requiring known stimulus timing. These inferred signals can be used for subsequent modeling of directed interactions between brain regions, making them relevant for future extensions toward effective connectivity estimation [66].

## Funding

This work was funded by the Radboud Excellence Initiative. Ioan Gabriel Bucur was funded through the Radboud Healthy Data program. The first dataset was provided by the Human Connectome Project, WU‐Minn Consortium (Principal Investigators: David Van Essen and Kamil Ugurbil; 1U54MH091657), funded by the 16 NIH Institutes and Centers that support the NIH Blueprint for Neuroscience Research; and by the McDonnell Center for Systems Neuroscience at Washington University.

## Conflicts of Interest

The authors declare no conflicts of interest.

## Data Availability

The data that support the findings of this study are available in the Human Connectome Project at https://www.humanconnectome.org/. These data were derived from the following resources available in the public domain: – HCP Young Adult, https://www.humanconnectome.org/study/hcp‐young‐adult 3mm‐preprocessed data from the IBC project, https://search.kg.ebrains.eu/instances/Dataset/ed615ee5‐fdaa‐4f1d‐8fcd‐8c55d05a4e2d.

## ENDNOTES

^1^
The time series image on the left is Figure 4.1 from Sidén [41], used under Creative Commons Attribution‐NonCommercial 4.0. The brain activation map is from https://upload.wikimedia.org/wikipedia/commons/4/4a/1206_FMRI.jpg.

## Appendix A

### Derivation of the Full Conditional Posterior for the MCMC Algorithm

The posterior distributions are based on the methods proposed by Sidén et al. [55] and Spencer et al. [29], and have been adapted to fit the structure of our model. The full conditional distributions for the model are derived through the following steps:

#### Likelihood

The likelihood can be expressed as

$$\begin{array}{cc} & p\left(Y|\beta ,A,\lambda \right)\propto \overset{S}{\underset{s=1}{\prod }}\overset{T}{\underset{t=P+1}{\prod }}\overset{R}{\underset{r=1}{\prod }}𝒩\\ & \;\left(y_{tr}^{s}-{\left(x_{tr,\cdot }^{s}\right)}^{⊤}\beta_{\cdot ,r}^{s};{\left({\tilde{y}}_{tr,\cdot }^{s}-{\tilde{X}}_{tr,\cdot }^{s}\;\beta_{\cdot ,r}^{s}\right)}^{⊤}a_{\cdot ,r}^{s},\frac{1}{\lambda_{r}^{s}}\right), \end{array}$$

where ${\tilde{X}}_{tr,\cdot }^{s}$ is a matrix P×K that contains the P rows of the design matrix before the time point t for subject s and region r. Note that the dimension of the regressors K is in fact K+1 because we are adding an intercept term, but in the rest of the appendix we will use K for simplicity and forgo the asterisk notation. Similarly, ${\tilde{y}}_{tr,\cdot }^{s}$ is a P×1 vector and contains the p values of the observed BOLD responses just before time point t. Note that we condition on the first P time points for simplicity. The nonconstant part of the log‐likelihood can be written as

$$\begin{array}{ll} \log p\left(Y|\cdot \right) & =\frac{T-P}{2}\overset{S}{\underset{s=1}{\sum }}\overset{R}{\underset{r=1}{\sum }}\log(\lambda_{r}^{s})-\frac{1}{2}\overset{S}{\underset{s=1}{\sum }}\overset{T}{\underset{t=P+1}{\sum }}\overset{R}{\underset{r=1}{\sum }}\\ & \;{\left[\left(y_{tr}^{s}-{\left(x_{tr,\cdot }^{s}\right)}^{⊤}\beta_{\cdot ,r}^{s}\right)-{\left({\tilde{y}}_{tr,\cdot }^{s}-{\tilde{X}}_{tr,\cdot }^{s}\;\beta_{\cdot ,r}^{s}\right)}^{⊤}a_{\cdot ,r}^{s}\right]}^{⊤}\lambda_{r}^{s}\\ & \cdot \left[\left(y_{tr}^{s}-{\left(x_{tr,\cdot }^{s}\right)}^{⊤}\beta_{\cdot ,r}^{s}\right)-{\left({\tilde{y}}_{tr,\cdot }^{s}-{\tilde{X}}_{tr,\cdot }^{s}\;\beta_{\cdot ,r}^{s}\right)}^{⊤}a_{\cdot ,r}^{s}\right]+\text{const}\\ & =\frac{T-P}{2}\overset{S}{\underset{s=1}{\sum }}\overset{R}{\underset{r=1}{\sum }}\log(\lambda_{r}^{s})-\frac{1}{2}\overset{S}{\underset{s=1}{\sum }}\overset{R}{\underset{r=1}{\sum }}\\ & \;{\left[\left(y_{\cdot ,r}^{s}-X_{r,\cdot }^{s}\;\beta_{\cdot ,r}^{s}\right)-{\left({\tilde{Y}}_{r,\cdot }^{s}-{\tilde{X}}_{r,\cdot }^{s}\;\beta_{\cdot ,r}^{s}\right)}^{⊤}a_{\cdot ,r}^{s}\right]}^{⊤}\lambda_{r}^{s}\\ & \cdot \left[\left(y_{\cdot ,r}^{s}-X_{r,\cdot }^{s}\;\beta_{\cdot ,r}^{s}\right)-{\left({\tilde{Y}}_{r,\cdot }^{s}-{\tilde{X}}_{r,\cdot }^{s}\;\beta_{\cdot ,r}^{s}\right)}^{⊤}a_{\cdot ,r}^{s}\right]+\text{const}. \end{array}$$

In the last expression, the sums and indexing with respect to t have been removed. Note that the size of ${\tilde{Y}}_{r,\cdot }^{s}$ is P×(T−P) and the size of ${\tilde{X}}_{r,\cdot }^{s}$ is P×(T−P)×K. In what follows, we carry out the matrix multiplications involving the 3D tensor ${\tilde{X}}_{r,\cdot }^{s}$ and other tensors over the appropriate dimension, but we do not state this explicitly. The log‐likelihood can now be rewritten as

$$\begin{array}{ll} \log p(Y|\cdot ) & =\frac{T-P}{2}\overset{S}{\underset{s=1}{\sum }}\overset{R}{\underset{r=1}{\sum }}\log(\lambda_{r}^{s})-\\ & \;-\frac{1}{2}\overset{S}{\underset{s=1}{\sum }}\overset{R}{\underset{r=1}{\sum }}\lambda_{r}^{s}\left[{\left(y_{\cdot ,r}^{s}\right)}^{⊤}y_{\cdot ,r}^{s}-2{\left(y_{\cdot ,r}^{s}\right)}^{⊤}X_{r,\cdot }^{s}\;\beta_{\cdot ,r}^{s}\right.\\ & \;+{\left(X_{r,\cdot }^{s}\;\beta_{\cdot ,r}^{s}\right)}^{⊤}X_{r,\cdot }^{s}\;\beta_{\cdot ,r}^{s}-2{\left(y_{\cdot ,r}^{s}\right)}^{⊤}{\tilde{Y}}_{r,\cdot }^{s}\;a_{\cdot ,r}^{s}+{\left({\tilde{Y}}_{r,\cdot }^{s}\;a_{\cdot ,r}^{s}\right)}^{⊤}{\tilde{Y}}_{r,\cdot }^{s}\;a_{\cdot ,r}^{s}\\ & \;+{\left(\beta_{\cdot ,r}^{s}\right)}^{⊤}B_{r}^{s}a_{\cdot ,r}^{s}+{\left(a_{\cdot ,r}^{s}\right)}^{⊤}B_{r}^{s}\beta_{\cdot ,r}^{s}\\ & \;-{\left(\beta_{\cdot ,r}^{s}\right)}^{⊤}\left(F_{r}^{s}a_{\cdot ,r}^{s}+{\left(F_{r}^{s}a_{\cdot ,r}^{s}\right)}^{⊤}\right)\beta_{\cdot ,r}^{s}-{\left(a_{\cdot ,r}^{s}\right)}^{⊤}\\ & \;\left.\left(D_{r}^{s}\beta_{\cdot ,r}^{s}+{\left(D_{r}^{s}\beta_{\cdot ,r}^{s}\right)}^{⊤}\right)a_{\cdot ,r}^{s}+{\left(\beta_{\cdot ,r}^{s}\right)}^{⊤}({\left(a_{\cdot ,r}^{s}\right)}^{⊤}G_{r}^{s}a_{\cdot ,r}^{s})\beta_{\cdot ,r}^{s}\right]+\text{const}, \end{array}$$

where

$$\begin{array}{lll} B_{r}^{s} & ={\left(y_{\cdot ,r}^{s}\right)}^{⊤}{\tilde{X}}_{r,\cdot }^{s}+{\tilde{Y}}_{r,\cdot }^{s}X_{r,\cdot }^{s}\;\; & \text{is of size}\;P\times K,\\ F_{r}^{s} & ={\left(X_{r,\cdot }^{s}\right)}^{⊤}{\tilde{X}}_{r,\cdot }^{s}\;\; & \text{is of size}\;K\times K\times P,\\ D_{r}^{s} & ={\tilde{Y}}_{r,\cdot }^{s}{\tilde{X}}_{r,\cdot }^{s}\;\; & \text{is of size}\;P\times K\times P,\\ G_{r}^{s} & ={\left({\tilde{X}}_{r,\cdot }^{s}\right)}^{⊤}{\tilde{X}}_{r,\cdot }^{s}\;\; & \text{is of size}\;P\times K\times K\times P. \end{array}$$

#### Full Conditional Posterior of $\beta^{s}$

$$\begin{array}{ll} \log p(\beta^{s}|Y,\cdot ) & =-\frac{1}{2}{\left(\beta_{\cdot ,r}^{s}\right)}^{⊤}{\tilde{W}}^{s}\beta_{\cdot ,r}^{s}+{\left(b_{\beta }^{s}\right)}^{⊤}\beta_{\cdot ,r}^{s}+\text{const},\\ & b_{\beta }^{s}=\underset{r\in \left\{1,2,\ldots ,R\right\}}{\mathrm{vec}}\left[\lambda_{r}^{s}\left({\left(y_{\cdot ,r}^{s}\right)}^{⊤}{\tilde{X}}_{r,\cdot }^{s}\right.\right.\left.\left.-{\left(a_{\cdot ,r}^{s}\right)}^{⊤}B_{r}^{s}+{\left(a_{\cdot ,r}^{s}\right)}^{⊤}D_{r}^{s}a_{\cdot ,r}^{s}\right)\right],\\ & {\tilde{W}}^{s}=\underset{r\in \left\{1,2,\ldots ,R\right\}}{\mathrm{blkdiag}}\left[\lambda_{r}^{s}\left({\left(X_{r,\cdot }^{s}\right)}^{⊤}X_{r,\cdot }^{s}-F_{r}^{s}a_{\cdot ,r}^{s}\right.\right.\\ & \;\left.\left.-{\left(F_{r}^{s}a_{\cdot ,r}^{s}\right)}^{⊤}+{\left(a_{\cdot ,r}^{s}\right)}^{⊤}G_{r}^{s}a_{\cdot ,r}^{s}\right)\right]+\mathrm{diag}(\alpha^{s})⊗Q_{\beta }^{s}, \end{array}$$

where $\mathrm{diag}(\alpha^{s})$ is a diagonal matrix containing the elements of the vector $\alpha^{s}$ and where ${\tilde{W}}^{s}=\underset{r\in \left\{1,2,\ldots ,R\right\}}{\mathrm{blkdiag}}[{{\tilde{W}}^{s}}_{r}]$ is a KR×KR block diagonal matrix with the K×K matrix ${{\tilde{W}}^{s}}_{r}$ as the $r^{\text{th}}$ block. The symbol ⊗ denotes the outer product. We thus have

$$\beta_{\cdot ,r}^{s}|Y,\cdot ∼𝒩\left({\left({\tilde{W}}^{s}\right)}^{-1}b_{\beta }^{s},{\left({\tilde{W}}^{s}\right)}^{-1}\right).$$

#### Full Conditional Posterior of $a_{\cdot ,r}^{s}$

$$\begin{array}{ll} \log p\left(a_{\cdot ,r}^{s}|Y,\cdot \right) & =-\frac{1}{2}{\left(a_{\cdot ,r}^{s}\right)}^{⊤}{\tilde{J}}^{s}a_{\cdot ,r}^{s}+{\left(b_{a}^{s}\right)}^{⊤}a_{\cdot ,r}^{s}+\mathrm{const},\\ b_{a}^{s} & =\underset{r\in \left\{1,2,\ldots ,R\right\}}{\mathrm{vec}}\left[\lambda_{r}^{s}\left({\left({\tilde{Y}}_{r,\cdot }^{s}y_{\cdot ,r}^{s}\right)}^{⊤}\right.\right.\left.\left.-{\left(\beta_{\cdot ,r}^{s}\right)}^{⊤}B_{r}^{s}+{\left(\beta_{\cdot ,r}^{s}\right)}^{⊤}F_{r}^{s}\beta_{\cdot ,r}^{s}\right)\right]\\ {\tilde{J}}^{s} & =\underset{r\in \left\{1,\ldots ,R\right\}}{\mathrm{blkdiag}}\left[\lambda_{r}^{s}\left({\tilde{Y}}_{r,\cdot }^{s}{\left({\tilde{Y}}_{r,\cdot }^{s}\right)}^{⊤}\right.\right.\\ & \;\left.\left.-D_{r}^{s}\beta_{\cdot ,r}^{s}-{\left(D_{r}^{s}\beta_{\cdot ,r}^{s}\right)}^{⊤}+{\left(\beta_{\cdot ,r}^{s}\right)}^{⊤}G_{r}^{s}\beta_{\cdot ,r}^{s}\right)\right]\\ & \;+\mathrm{diag}(\tau^{s})⊗Q_{a}^{s}, \end{array}$$

where $\mathrm{diag}(\tau^{s})$ is a diagonal matrix containing the elements of the vector $\tau^{s}$ and where ${\tilde{J}}^{s}=\underset{r\in \left\{1,2,\ldots ,R\right\}}{\mathrm{blkdiag}}[{{\tilde{J}}^{s}}_{r}]$ is a PR×PR block diagonal matrix with the P×P matrix ${{\tilde{J}}^{s}}_{r}$ as the $r^{\text{th}}$ block. The symbol ⊗ denotes the outer product. We thus have

$$a_{\cdot ,r}^{s}|Y,\cdot ∼𝒩\left({\left({\tilde{J}}^{s}\right)}^{-1}b_{a}^{s},{\left({\tilde{J}}^{s}\right)}^{-1}\right).$$

#### Full Conditional Posterior of $\lambda^{s}$

$$\begin{array}{ll} \log p(\lambda^{s}|Y,\cdot ) & =({ū}_{2}-1)\overset{R}{\underset{r=1}{\sum }}\log(\lambda_{r}^{s})-\overset{R}{\underset{r=1}{\sum }}\frac{\lambda_{r}^{s}}{{ū}_{1r}^{s}}+\mathrm{const}\;\;\\ \frac{1}{{ū}_{1r}^{s}} & =\frac{1}{u_{1}}+\frac{1}{2}\left[{\left(y_{\cdot ,r}^{s}\right)}^{⊤}y_{\cdot ,r}^{s}-2{\left(y_{\cdot ,r}^{s}\right)}^{⊤}X_{r,\cdot }^{s}\beta_{\cdot ,r}^{s}\right.\\ & \;+{\left(X_{r,\cdot }^{s}\beta_{\cdot ,r}^{s}\right)}^{⊤}X_{r,\cdot }^{s}\beta_{\cdot ,r}^{s}-2{\left({\tilde{Y}}_{r,\cdot }^{s}y_{\cdot ,r}^{s}\right)}^{⊤}a_{\cdot ,r}^{s}\\ & \;+{\left(a_{\cdot ,r}^{s}\right)}^{⊤}{\tilde{Y}}_{r,\cdot }^{s}{\left({\tilde{Y}}_{r,\cdot }^{s}\right)}^{⊤}a_{\cdot ,r}^{s}+{\left(B_{r}^{s}\beta_{\cdot ,r}^{s}\right)}^{⊤}a_{\cdot ,r}^{s}+{\left(a_{\cdot ,r}^{s}\right)}^{⊤}B_{r}^{s}\beta_{\cdot ,r}^{s}\\ & \;-{\left(\beta_{\cdot ,r}^{s}\right)}^{⊤}\left(F_{r}^{s}a_{\cdot ,r}^{s}+{\left(F_{r}^{s}a_{\cdot ,r}^{s}\right)}^{⊤}\right)\beta_{\cdot ,r}^{s}\\ & \;-{\left(a_{\cdot ,r}^{s}\right)}^{⊤}\left(D_{r}^{s}\beta_{\cdot ,r}^{s}+{\left(D_{r}^{s}\beta_{\cdot ,r}^{s}\right)}^{⊤}\right)a_{\cdot ,r}^{s}\\ & \;\left.+{\left(\beta_{\cdot ,r}^{s}\right)}^{⊤}\left({\left(a_{\cdot ,r}^{s}\right)}^{⊤}G_{r}^{s}a_{\cdot ,r}^{s}\right)\beta_{\cdot ,r}^{s}\right]\;\;\\ {ū}_{2} & =\frac{T-P}{2}+u_{2}, \end{array}$$

so $\lambda_{r}^{s}|Y,\cdot ∼𝒢({ū}_{1r}^{s},{ū}_{2})$ for each region r and subject s.

#### Full Conditional Posterior of $\alpha^{s}$

$$\begin{array}{ll} \log p(\alpha^{s}|\cdot ) & =\left({\bar{q}}_{2}-1\right)\overset{S}{\underset{s=1}{\sum }}\overset{K}{\underset{k=1}{\sum }}\log(\alpha_{k}^{s})-\overset{S}{\underset{s=1}{\sum }}\overset{K}{\underset{k=1}{\sum }}\frac{\alpha_{k}^{s}}{{\bar{q}}_{1k}^{s}}+\mathrm{const},\\ \frac{1}{{\bar{q}}_{1k}^{s}} & =\frac{1}{2}\beta_{k,\cdot }^{s}Q_{\beta }^{s}{\left(\beta_{k,\cdot }^{s}\right)}^{⊤}+\frac{1}{q_{1}},\\ {\bar{q}}_{2} & =\frac{R}{2}+q_{2}, \end{array}$$

so $\alpha_{k}^{s}|\cdot ∼𝒢\left({\bar{q}}_{1k}^{s},{\bar{q}}_{2}\right)$ for each regressor k and subject s.

#### Full Conditional Posterior of $\tau^{s}$

$$\begin{array}{ll} \log p(\tau^{s}|\cdot ) & =({\bar{v}}_{2}^{s}-1)\overset{S}{\underset{s=1}{\sum }}\overset{P}{\underset{p=1}{\sum }}\log(\tau_{p}^{s})-\overset{S}{\underset{s=1}{\sum }}\overset{P}{\underset{p=1}{\sum }}\frac{\tau_{p}^{s}}{{\bar{v}}_{1p}^{s}}+\mathrm{const},\;\;\;\\ \frac{1}{{\bar{v}}_{1p}^{s}} & =\frac{1}{2}a_{p,\cdot }^{s}Q_{a}^{s}{\left(a_{p,\cdot }^{s}\right)}^{⊤}+\frac{1}{v_{1}},\\ {\bar{v}}_{2} & =\frac{R}{2}+v_{2}, \end{array}$$

so $\tau_{p}^{s}|\cdot ∼𝒢({\bar{v}}_{1p}^{s},{\bar{v}}_{2})$ for each order p and subject s.

#### Block Gibbs Sampling for Precision Matrix Ω

To sample the precision matrix Ω using block Gibbs sampling as outlined in [36], we update Ω one column and row at a time. Let $H=(\eta_{kl})$ be a R×R symmetric matrix with zeros in the diagonal entries and η in the upper diagonal entries. We proceed iteratively over each column (and row, due to symmetry) of Ω, conditioning on the rest of the matrix and latent variables. Given the initial value of $\beta_{K}$, the shrinkage parameter ζ, the latent variables H, and the initial value of the precision matrix Ω, we update Ω as follows:

1. Compute the cross‐product matrix:
  $$N=\beta_{K}{\left(\beta_{K}\right)}^{⊤}$$
2. For each row/column r=1,2,…,R, sample δ from the following distribution:
  $$\delta ∼𝒢\left(\frac{S}{2}+1,\;\frac{N_{rr}+\zeta }{2}\right)$$
3. Sample the vector ν of size R×1 from the following distribution:
  ν∼𝒩(μ,V),
  where μ,V are defined as
  $$V={\left((N_{rr}+\zeta ){\left(\Omega_{-r,-r}\right)}^{-1}+{\left(\mathrm{diag}(H_{-r,r})\right)}^{-1}\right)}^{-1}$$
  $$\mu =-V{\left(N_{r,-r}\right)}^{⊤}$$
4. Update off‐diagonal elements:
  $$\Omega_{r,-r}=\Omega_{-r,r}=\nu$$
5. Update diagonal element:
  $$\omega_{rr}=\delta +\nu^{⊤}{\left(\Omega_{r,r}\right)}^{-1}\nu$$
6. For all $r<r^{\prime }\leq R$, sample:
  $$\frac{1}{\eta_{rr^{\prime }}}∼\text{Inverse‐Gaussian}\left(\sqrt{\frac{\zeta^{2}}{\omega_{rr^{\prime }}^{2}}},\;\zeta^{2}\right)$$
7. Draw ζ from a $𝒢(\bar{g_{1}},\bar{g_{2}})$ distribution where
  $$\bar{g_{1}}=g_{1}+\frac{R(R+1)}{2}\;\text{and}\;\bar{g_{2}}=g_{2}+\frac{\sum_{i}\sum_{j}|\omega_{ij}|}{2}$$

This procedure is repeated for all rows/columns of Ω to generate a complete update in each iteration of the Gibbs sampler.
